# Expanding the World of Marine Bacterial and Archaeal Clades

**DOI:** 10.3389/fmicb.2015.01524

**Published:** 2016-01-08

**Authors:** Pelin Yilmaz, Pablo Yarza, Josephine Z. Rapp, Frank O. Glöckner

**Affiliations:** ^1^Microbial Genomics and Bioinformatics Research Group, Max Planck Institute for Marine MicrobiologyBremen, Germany; ^2^Ribocon GmbHBremen, Germany; ^3^HGF-MPG Joint Research Group for Deep Sea Ecology and Technology, Max Planck Institute for Marine Microbiology, Bremen and the Alfred Wegener Institute Helmholtz Centre for Polar and Marine ResearchBremerhaven, Germany; ^4^Life Sciences and Chemistry, Jacobs UniversityBremen, Germany

**Keywords:** marine, bacterioplankton, bacterial phylogeny, bacterial taxonomy, ecology, rare taxa

## Abstract

Determining which microbial taxa are out there, where they live, and what they are doing is a driving approach in marine microbial ecology. The importance of these questions is underlined by concerted, large-scale, and global ocean sampling initiatives, for example the International Census of Marine Microbes, Ocean Sampling Day, or Tara Oceans. Given decades of effort, we know that the large majority of marine Bacteria and Archaea belong to about a dozen phyla. In addition to the classically culturable Bacteria and Archaea, at least 50 “clades,” at different taxonomic depths, exist. These account for the majority of marine microbial diversity, but there is still an underexplored and less abundant portion remaining. We refer to these hitherto unrecognized clades as *unknown*, as their boundaries, names, and classifications are not available. In this work, we were able to characterize up to 92 of these *unknown* clades found within the bacterial and archaeal phylogenetic diversity currently reported for marine water column environments. We mined the SILVA 16S rRNA gene datasets for sequences originating from the marine water column. Instead of the usual subjective taxa delineation and nomenclature methods, we applied the candidate taxonomic unit (CTU) circumscription system, along with a standardized nomenclature to the sequences in newly constructed phylogenetic trees. With this new phylogenetic and taxonomic framework, we performed an analysis of ICoMM rRNA gene amplicon datasets to gain insights into the global distribution of the new marine clades, their ecology, biogeography, and interaction with oceanographic variables. Most of the new clades we identified were interspersed by known taxa with cultivated members, whose genome sequences are available. This result encouraged us to perform metabolic predictions for the novel marine clades using the PICRUSt approach. Our work also provides an update on the taxonomy of several phyla and widely known marine clades as our CTU approach breaks down these randomly lumped clades into smaller objectively calculated subgroups. Finally, all taxa were classified and named following standards compatible with the Bacteriological Code rules, enhancing their digitization, and comparability with future microbial ecological and taxonomy studies.

## Introduction

In the rapidly changing and highly dynamic marine ecosystems, marine microbes act as the invisible “gatekeepers.” They inhabit all marine ecosystems, from the tropics to the polar waters and from well-lit surface waters to the deep abyss. They harvest and transduce solar energy—with an estimated contribution to global primary productivity of between 50 and 90% (Falkowski et al., 1998). They catalyze key biogeochemical transformations of all nutrients and trace elements that sustain the organic productivity of the ocean. Finally, they also represent a vast and dynamic reservoir of genetic variability that is yet to be tapped into. For many years, the study of marine microbes was hampered by the fact that the majority of microorganisms (90–99%) cannot be cultured under standard laboratory conditions. It was only with the development of a molecular toolbox to sequence DNA from the natural environment that information about the exceptional bacterial and archaeal diversity in the ocean began to accumulate (Pace et al., 1985; Amann et al., 1995; Karl, 2007). To date, perhaps thousands of research papers have been written on marine microbial diversity and communities. Nevertheless, determining which microbial taxa are out there, where they live, and what they are doing is still an important question in marine microbial ecology. The importance of these questions is underlined by concerted, large-scale, and global ocean sampling initiatives, for example the International Census of Marine Microbes (Amaral-Zettler et al., 2010), Global Ocean Sampling expedition (GOS) (Yooseph et al., 2007), Ocean Sampling Day (Kopf et al., 2015), Tara Oceans (Karsenti et al., 2011; Bork et al., 2015), or the Malaspina expedition (http://scientific.expedicionmalaspina.es/).

Given decades of effort, we now know that the large majority of known marine Bacteria and Archaea belong to a dozen bacterial and archaeal phyla. Furthermore, in addition to the classically culturable Bacteria and Archaea, at least 50 “clades,” at different taxonomic depths, exist (Fuhrman and Hagström, 2008). Originally, these clades were defined as clone sequences originating from one or two specific environments, and represented the “uncultivated” marine Bacteria and Archaea (Fuhrman and Hagström, 2008). With the development of improved cultivation methods, single-cell genomics, and finally genome assemblies from metagenomes, a lot more is now known about these clades in terms of their phylogeny, physiology, ecology, and metabolism. Despite the fact that, along with the cultivable Bacteria and Archaea, these marine clades account for the majority of the marine microbial diversity, there is still an underexplored, novel portion remaining. For example, a survey of GOS metagenomic reads containing 16S rRNA fragments revealed that, 4–5% of these fragments could not be taxonomically assigned to any known orders, while 20% could not be assigned to known families (Yilmaz et al., 2011b). Incidentally, it has been suggested that these unclassified sequences overlap with the “rare biosphere” (Sogin et al., 2006; Lynch et al., 2012). Rare and low-abundant taxa are often not captured by cultivation, or by environmental sequencing, and their ecology and metabolic roles therefore remain poorly understood. While it is possible that rare taxa may just represent negligible phylogenetic novelty, there is growing evidence that they often contribute to biogeochemical cycles, and can increase in abundance with changing conditions (Lynch and Neufeld, 2015). Another source of novelty for lower-rank taxa probably corresponds to unknown marine microbial symbionts, which due to sequencing preferences and methodologies still remain underrepresented in the databases.

Our aim in this work was to characterize part of the marine microbial diversity, specifically focusing on *unknown* clades, meaning those clades with unknown boundaries, names and classifications. In order to achieve this, we mined the SILVA 16S rRNA datasets for sequences originating from the marine water column. New phylogenetic trees were constructed for all phyla that contained members from marine origin. Aspects such as size, phylogenetic depth, or a standard nomenclature format of environmental clades are generally not considered during tree reconstruction, and few systematic surveys exist (Newton et al., 2011). To overcome this issue, we applied the candidate taxonomic unit (CTU) circumscription system, along with a standardized nomenclature (Yarza et al., 2014) to the sequences in these phylogenetic trees, instead of a subjective taxa delineation and nomenclature method. Due to the exhaustive manual curation undertaken for SILVA taxonomy, with the help of domain experts (Yilmaz et al., 2014), almost all major *known* marine clades have been annotated in the original SILVA guide tree. By mapping the sequences from this guide tree to the new trees, and marking all clades that contained sequences from known marine clades, we uncovered which clades had remained as *unknown*.

With this new phylogenetic and taxonomic framework, we performed a large-scale meta-analysis of publicly available 16S rRNA amplicon datasets (specifically ICoMM marine water samples) to gain insights into the global distribution of newly recognized marine clades, their ecology, biogeography, and interaction with oceanographic variables. ICoMM, which represents a first inventory of marine microbial diversity and biogeography based on rRNA gene data, is an ideal dataset for this analysis—it is based on a single 16S rRNA region, a standardized experimental setup was used for all samples, and most importantly, it contains rich contextual metadata (Yilmaz et al., 2011a).

Most of the new clades we identified were interspersed by known taxa with cultivated members, whose genome sequences are available. This result encouraged us to perform metabolic predictions for these clades using the PICRUSt approach (Langille et al., 2013). PICRUSt is designed to predict the functional composition of metagenomes using marker gene data (such as rRNA) and a database of reference genomes. Specifically, an ancestral-state reconstruction algorithm predicts which gene families are possibly present. We acknowledge that phylogeny and function are at best imperfectly correlated, however the original PICRUSt paper, along with several others have demonstrated that 16S rRNA based phylogenetic trees mirror functional gene clusters (Martiny et al., 2013; Aßhauer et al., 2015). We present these predictions not as the ground-truth, but as a possibility for these clades, given their habitat and geographical distribution that we determined based on the ICoMM dataset.

## Materials and methods

### Phylogenetic analysis and construction of taxonomic framework

The SILVA SSU Ref 111 dataset served as a basis for all further analysis. This dataset contains 739,633 high-quality and nearly full−length (>900 bp for *Archaea*; >1200 bp for *Bacteria*) sequences of the SSU rRNA gene. All sequences in the SILVA database are aligned and quality controlled (Quast et al., 2013). By using a complex regular expression, a keyword search was performed to extract all sequences from the SILVA SSU Ref 111, for which the isolation source could be traced back to the marine water column (Table S1). Further, based on organism name and strain field metadata, we identified sequences originating from environmental sequences, and not from cultivated organisms. Once all sequences that fit to these criteria were identified, they were inspected in the context of the clades that they belonged to in the SILVA guide tree. Clades that consisted of at least 50% marine water column sequences, but were mixed with other marine-source sequences (sediment, hydrothermal vent etc.), were still marked entirely for further tree reconstruction. Approximately 45,000 starting sequences were then filtered for the tree reconstruction process based on the following criteria; minimum Pintail value of 75, minimum sequence quality of 75, and a minimum alignment quality of 90. Phylogenetic trees were calculated individually for each phylum, and an outgroup of at least five cultivated or type strain organism sequences from a neighboring phylum were selected for each tree. Prior to tree calculation, alignment positions were filtered with a 10% base conservation filter that was calculated with all sequences of each phylum. Maximum Likelihood tree reconstructions were performed with RAxML version 7.0.4 (Stamatakis, 2006) with 100 rapid bootstrap inferences and a thorough maximum likelihood search, using the GAMMA evolutionary model with GTR correction. We chose the CTU recognition process as proposed by Yarza et al. (2014) in order to construct a unified taxonomic framework. To generate OTUs, we performed a hierarchical furthest neighbor clustering using MOTHUR (Schloss et al., 2009) (v1.20.3) with specific sequence identity thresholds for each taxonomic rank level (75% Phylum, 78.5% Class, 82% Order, 86.5% Family, and 94.5% Genus). CTUs were constructed by combining the tree topology with the OTU information in order to form monophyletic clades. All sequences used in tree reconstruction and CTU recognition are available as a fasta file, as well as an arb file with phylogenetic trees under http://www.arb-silva.de/download/archive/marine_taxa/.

### Analysis and contextual annotation of ICoMM samples

Trimmed fasta files of all reads from 513 water column samples from the ICoMM project, plus several MIRADA LTER project samples were selected and downloaded from https://vamps.mbl.edu/ on July 10 2013. All sequence reads were then processed with the next generation sequencing analysis pipeline of the SILVA project (SILVAngs 1.0) (Klindworth et al., 2013; Quast et al., 2013). To ensure classification accuracy despite the short read length of the ICoMM data (average 60 bases), the taxonomic rank “order” was selected for further analyses.

We used the World Ocean Atlas 2005 (WOA05) (Antonov et al., 2006; Garcia et al., 2006a,b; Locarnini et al., 2006) interpolator function of megx.net portal (Kottmann et al., 2010) to complement or extend the available ICoMM contextual data as described previously (Yilmaz et al., 2012). Furthermore, we simplified the existing Environment Ontology (Buttigieg et al., 2013) material terms for each sample, and complemented these terms with a qualitative assessment of oceanic primary productivity (low, mid, high) based on WOA05 chlorophyll *a* concentrations and Longhurst province descriptions (Table S3) (Longhurst, 2010).

### PICRUSt and other statistical analyses

All statistical analyses, and further visualization of the data was performed in R statistical computing environment (v3.0.2). Indicator species and site association analyses were performed with package indicspecies (v1.7.4) (De Caceres and Legendre, 2009). The correlations between clade relative abundance and oceanographic variables were calculated with package Hmisc (v 3.12-2) and using spearman rank correlations. PICRUSt (v0.9.2) predictions (Langille et al., 2013) were performed based on the SILVA SSU Ref111 dataset. As this is a deviation from the normal usage of the PICRUSt tool with an amplicon dataset with different samples, we designated each bacterial and archaeal phylum name a “sample” and simply treated all sequences of this phylum as sequences within a sample. The relative abundances were not used for interpretation, and only the presence or absence of a certain enzyme was considered.

## Results and discussion

### Overview of phylogenetic reconstruction, taxonomic framework, and ICoMM samples analysis

In total, 11 phylogenetic trees were calculated, covering Acidobacteria, Actinobacteria (and neighbors), Bacteroidetes (and neighbors), Chloroflexi, Deferribacteres, Verrucomicrobia, Planctomycetes, Lentisphaerae, Euryarchaeota, Thaumarchaeota, and the classes of *Alpha*-, *Beta-, Gamma-* and Deltaproteobacteria. The number of marine sequences within each of these phyla is given in Table S2. By ordinary CTU analysis, these phyla constituted 42 candidate classes, 148 candidate orders, 336 candidate families, and 1008 candidate genera with at least two sequences. Numbers of lower-rank groups within phyla or classes were highly variable, and they did not necessarily relate to the number of sequences within a group, but they related rather to the diversity within that group. For example, Verrucomicrobia only had 321 sequences associated, and Thaumarcheota was at least 20-fold larger, but the former contained around half as much genera as Thaumarcheota (Table S2).

As outlined in the methods section, we used the new phylogeny and the taxonomic framework as the taxonomic classification reference basis for selected ICoMM projects. Here, we analyzed 513 publicly available samples from the ICoMM project (Table S3; Figure S1) using the SILVAngs pipeline.

Major known clades were already annotated in the SILVA guide tree, therefore they could be identified in the new phylogenetic trees by simply marking all sequences belonging to these clades (Table S2). After identifying and fishing out known clades from the CTU taxonomic hierarchy, 9335 sequences out of 45,000 initial sequences, in 92 orders, remained for further analysis. This may either mean that most marine microbial sequences belong to a few clades, or that the richness is much higher, but is obscured by the abundant and preferred lineages. The relative sequence abundances of the new clades did not change much with respect to the total community pictures, though the less abundant phyla, i.e., *Planctomycetes, Verrucomicrobia*, became relatively more dominant, while archaeal phyla almost completely disappeared (Figure S2), as the majority of the archaeal sequences did belong to known marine archaeal taxa.

### Archaea

#### Phylogeny and taxonomy

**Figure 1:**
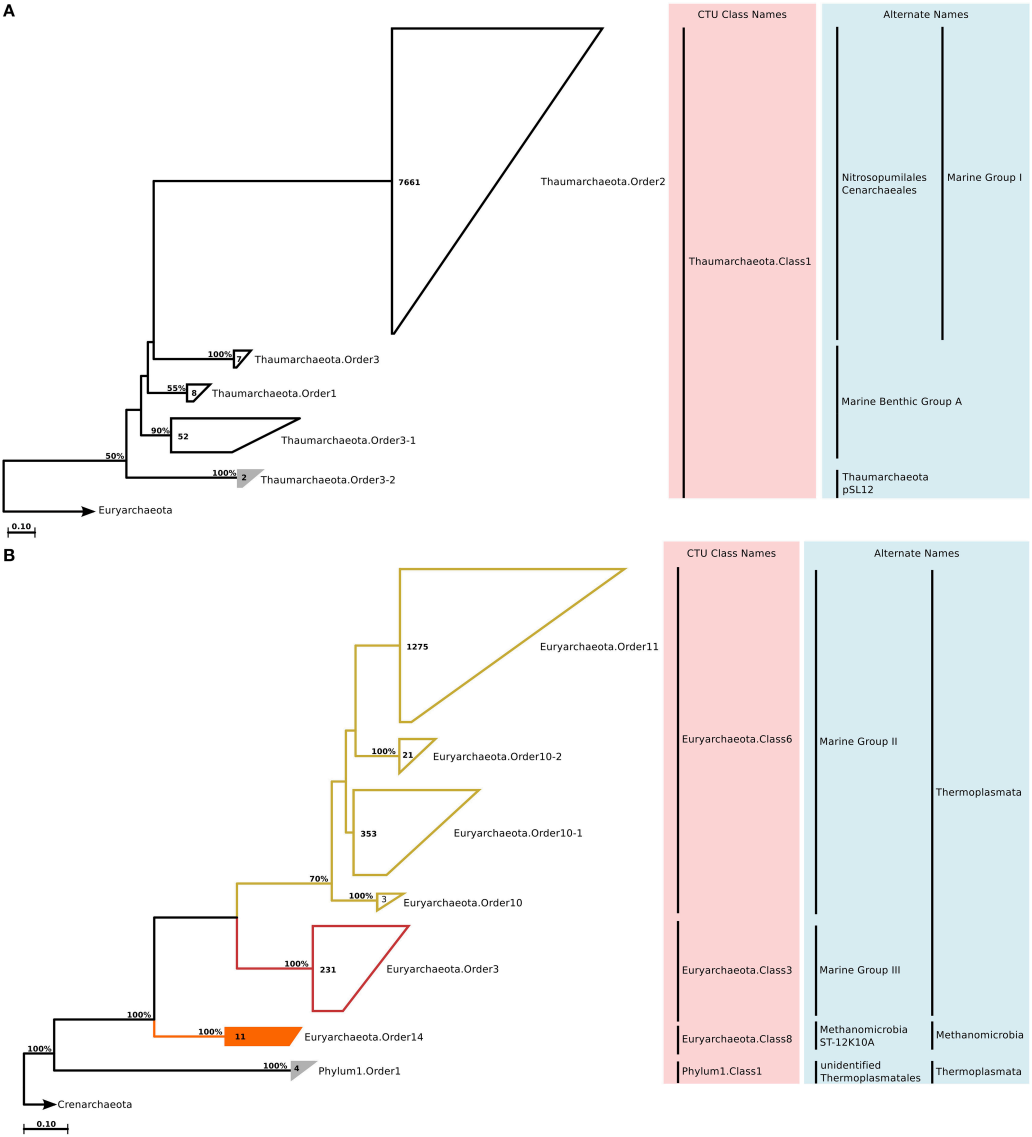
**Phylogenetic trees illustrating the relationships among SSU rRNA gene sequences belonging to uncultured marine Thaumarchaeota (A) and Euryarchaeota (B)**. The tree is displayed at order level (minimum 82% sequence identity). Colored branches and annotations in the light pink box next to the tree indicate different CTU classes. Annotations in the light blue box indicate alternate or currently used taxa names at different rank levels. Newly recognized clades are groups filled with solid color, whereas unfilled groups indicate known clades. Bootstrap support for topologies was calculated with 100 repetitions, and only values above 50% are shown on the branches. The numbers inside the groups indicate the number of sequences in that group. The outgroup sequences are indicated by an arrow. Bar = nucleotide changes per site.

Studies on marine Archaea recognize three major groups that dominate the marine water column environments; Marine Group I of *Thaumarchaeota*, and Marine Groups II and III of *Euryarchaeota* (DeLong, 1998). Two sequences that could not be assigned to any of these groups formed a small clade named *Thaumarchaeota*.Order3-2, which by sequence identity values was mostly related to Marine Benthic Group A (Figure 1A). Among the marine euryarchaeal sequences, the only new clades that we could identify were two small clusters named *Euryarchaeota*.Order14 (within *Methanomicrobia*), and Phylum1.Order1 (Figure 1B). Interestingly, sequences that constituted Phylum1.Order1 were part of Marine Group III in the original SILVA Ref guide tree, but made up an entirely new phylum of Archaea in the newly constructed tree as suggested by the deep branching and distance to other sequences.

#### Habitat

**Figure 2:**
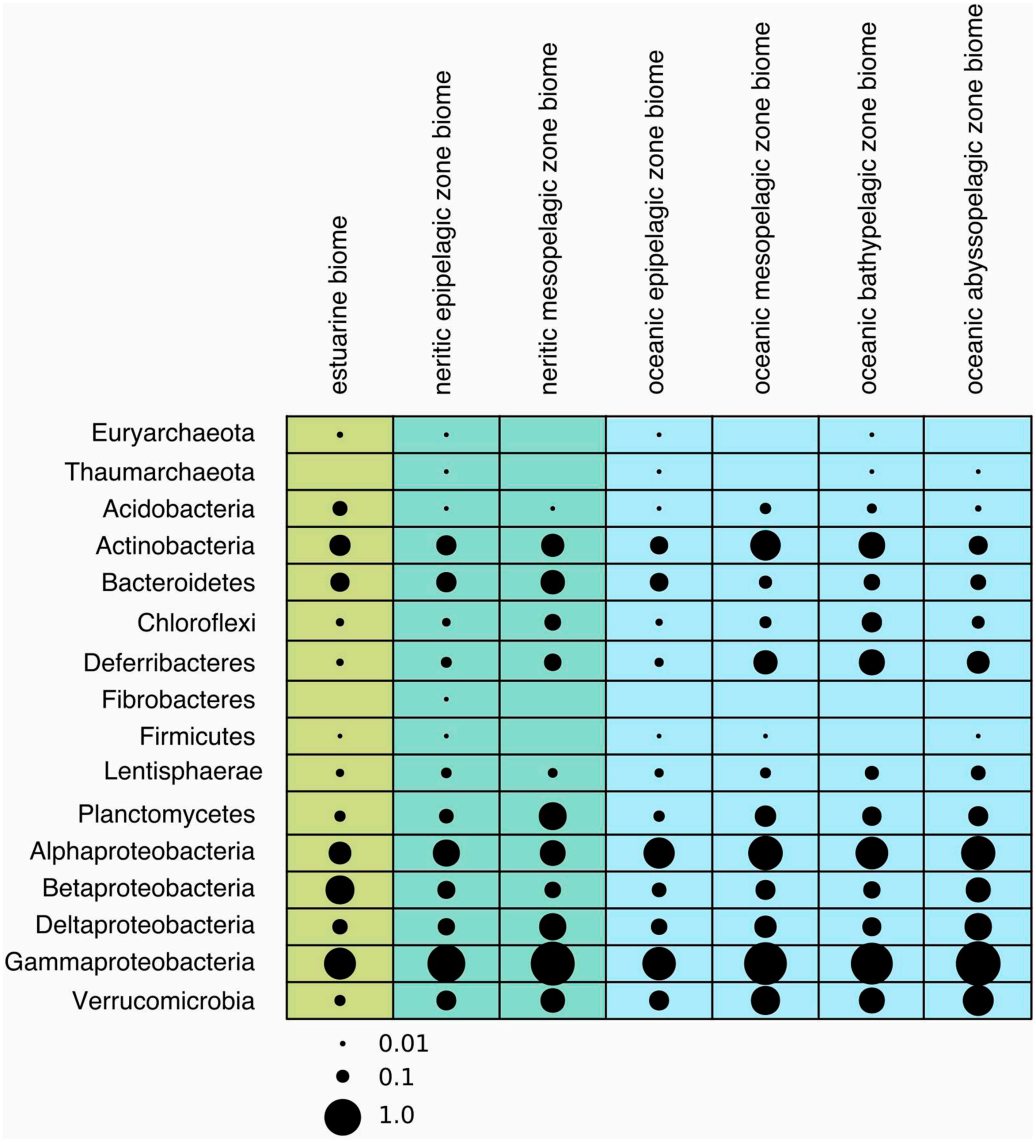
**Bubble plot showing the average relative abundances of newly recognized clades in different phyla across biomes**. The scale for bubbles are indicated under the plot, and values were scaled from 0 to 1, with 0 representing the minimum average relative abundance, and 1 representing the maximum relative abundance.

**Figure 3:**
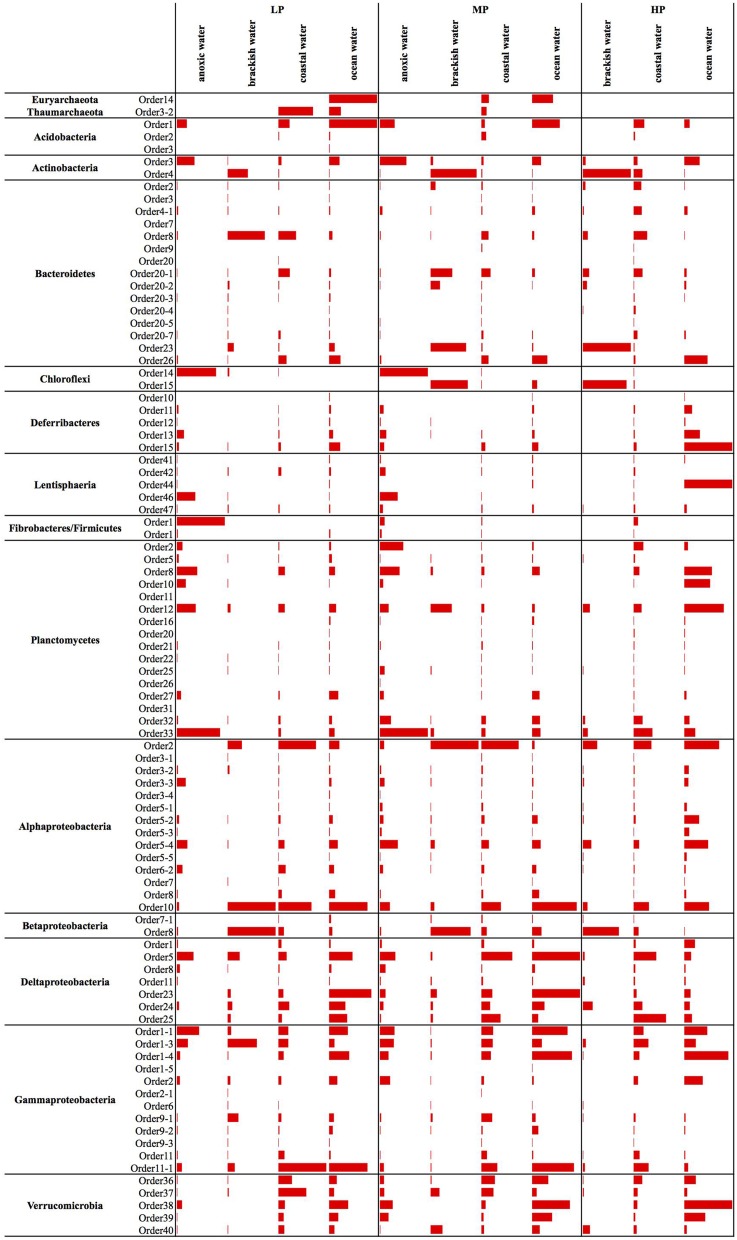
**Panel figure showing the average relative abundances of all new candidate orders across different water body types**. The bars for each phylum are scaled according to the values within that phylum. To increase visibility of the bars, the relative abundance values are given separately in a table (Table S4). LP, low productivity; MP, mid productivity; HP, high productivity.

**Figure 4:**
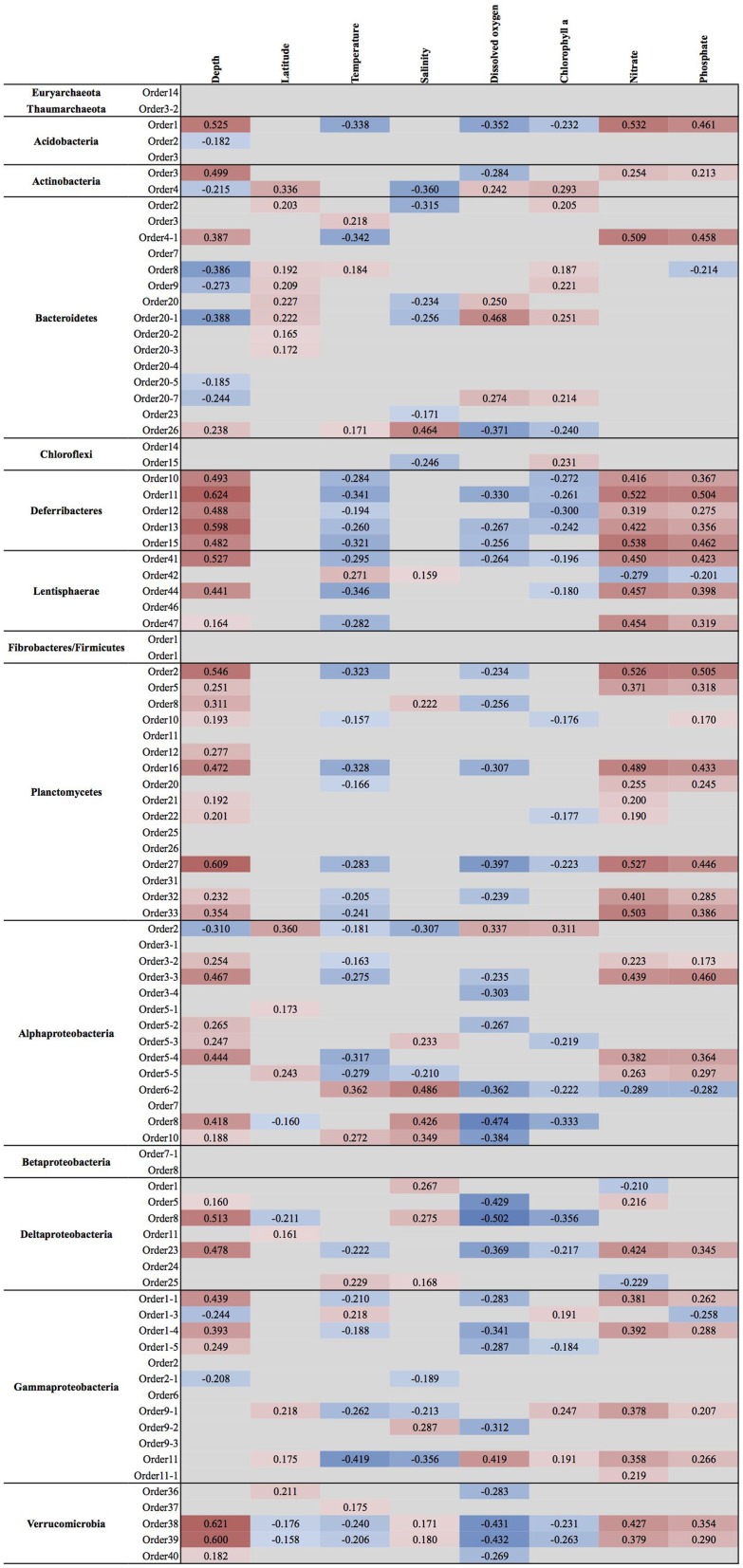
**Spearman rank correlation coefficients between clade relative abundance and available physicochemical parameters**. Only correlations that had a *p*-value of less than 0.05 are shown. Cells are colored with hues of red and blue to indicate the strength of positive or negative correlation coefficient, respectively.

Earlier studies on marine Archaea suggested that *Thaumarchaeota* are particularly abundant in the deep sea and average about 20–40% of all bacterioplankton at depths below a few hundred meters (Fuhrman and Ouverney, 1998; DeLong et al., 1999; Herndl et al., 2005). *Euryarchaeota* are much less abundant in deep waters, but sometimes surpass *Thaumarchaeota* abundance in surface waters, especially during summer months (Iverson et al., 2012). Further, they are also found in association with other marine organisms, particularly eukaryotes such as corals and sponges (Muller et al., 2010). The only clades that we could identify in the ICoMM samples belonged to *Thaumarchaeota*.Order3-2 and *Euryarchaeota*.Order14. *Thaumarchaeota*.Order3-2 was present in coastal and open ocean surface waters, as well as in open oceans below depths of 1000 m. *Euryarchaeota*.Order14 was present in surface waters, but also in the bathypelagic zone (Figure 2). In terms of type of water bodies, we observed Euryarchaeota in low-to-mid productivity oceanic and coastal waters. The *Thaumarcheota* distribution was essentially the same, but with lower relative abundances (Figure 3). However, neither indicator species nor site association tests returned any significant results (Table 1; Table S4), as well as correlations with oceanographic variables (Figure 4). Notably, the original clone sequences that constituted these clades were coming from the deep oceans (~1000 m), and for the euryarchaeal group, the clone sequences came exclusively from brine-seawater interfaces of hydrothermal brine systems. It is possible that these two groups represent very endemic and habitat specific groups.

**Table 1:** **Significant associations between different environments and relative abundance of newly recognized marine clades with a *p*-value of less than 0.05**.

**Clade**	**Environment**	***P*-value**
*Acidobacteria*.Order1	LP ocean water	0.010934285
*Actinobacteria.*Order3	MP anoxic water	0.010934285
*Alphaproteobacteria.*Order3-3	LP anoxic water	0.010934285
*Alphaproteobacteria.*Order3-4	LP ocean water	0.032477437
*Alphaproteobacteria.*Order6-2	LP coastal water	0.010934285
*Alphaproteobacteria.*Order8	LP ocean water	0.010934285
*Alphaproteobacteria.*Order10	LP ocean water	0.010934285
*Bacteroidetes.*Order2	HP coastal water	0.032477437
*Bacteroidetes.*Order9	MP coastal water	0.021759772
*Bacteroidetes.*Order20-2	MP brackish water	0.043088246
*Bacteroidetes.*Order20-4	HP coastal water	0.010934285
*Bacteroidetes.*Order20-7	HP coastal water	0.010934285
*Bacteroidetes.*Order26	LP ocean water	0.010934285
*Betaproteobacteria.*Order7-1	LP ocean water	0.010934285
*Deferribacteres.*Order10	LP ocean water	0.010934285
*Deferribacteres.*Order11	HP ocean water	0.032477437
*Deferribacteres.*Order12	LP ocean water	0.010934285
*Deferribacteres.*Order13	LP anoxic water	0.043088246
*Deferribacteres.*Order15	LP ocean water	0.010934285
*Deltaproteobacteria.*Order1	HP ocean water	0.010934285
*Deltaproteobacteria.*Order5	MP ocean water	0.010934285
*Deltaproteobacteria.*Order23	LP ocean water	0.010934285
*Gammaproteobacteria.*Order1-1	MP ocean water	0.010934285
*Gammaproteobacteria.*Order1-4	LP ocean water	0.010934285
*Gammaproteobacteria.*Order1-5	MP ocean water	0.043088246
*Gammaproteobacteria.*Order9-1	MP coastal water	0.010934285
*Gammaproteobacteria.*Order9-2	MP ocean water	0.010934285
*Gammaproteobacteria.*Order11-1	LP coastal water	0.043088246
*Lentisphaerae.*Order41	MP ocean water	0.043088246
*Lentisphaerae.*Order44	HP ocean water	0.010934285
*Lentisphaerae.*Order47	HP ocean water	0.010934285
*Planctomycetes.*Order16	MP ocean water	0.010934285
*Planctomycetes.*Order22	LP ocean water	0.032477437
*Planctomycetes.*Order27	LP ocean water	0.010934285
*Verrucomicrobia.*Order36	LP coastal water	0.010934285
*Verrucomicrobia.*Order37	LP coastal water	0.010934285
*Verrucomicrobia.*Order38	LP ocean water	0.010934285
*Verrucomicrobia.*Order39	LP ocean water	0.010934285

LP, low productivity; MP, mid productivity; HP, high productivity.

#### Metabolism predictions

Recent metagenomic, biogeochemical, and microbiological studies revealed the capacity of Marine Group I to oxidize ammonia, thus linking this abundant microbial clade to one of the key steps of the global nitrogen cycle (Walker et al., 2010; Pester et al., 2011). Marine Group II is photoheterotrophic (Iverson et al., 2012), and it was also shown that planktonic Archaea might take up amino acids (Ouverney and Fuhrman, 2000; Herndl et al., 2005).

PICRUSt (Table S6) returned a predicted nitrogenase (nifH) and an assimilatory nitrite reductase for *Euryarchaeota*.Order14, and a nitrate reductase involved in dissimilatory nitrate reduction (DNRA) for *Thaumarchaeota*.Order3-2. *Thaumarchaeota*.Order3-2 was predicted to harbor a carbon monoxide dehydrogenase (CODH), as well as a 4- hydroxybutanoyl-CoA dehydratase (abfD) of the 3-hydroxypropionate pathway. Both predictions are indicative of carbon fixation in this order. For *Euryarchaeota*.Order14, a large chain of ribulose-bisphosphate carboxylase (RuBisCo) was predicted. In terms of nutrient acquisition, complex and simple carbohydrate degradation (alpha-amylase, endoglucanase, beta-glucosidase) capabilities for *Euryarchaeota*.Order14, along with several sulfatases were predicted. Lastly, both orders were predicted to possess aminopeptidases and amidases, phosphate and iron transport systems. Previous reports show that *Archaea* can be autotrophs, mixotrophs or heterotrophs (Ingalls et al., 2006; Agogué et al., 2008; Hansman et al., 2009; Alonso-Sáez et al., 2012), which can potentially use bicarbonate (Könneke et al., 2005; Pitcher et al., 2011), amino acids (Ouverney and Fuhrman, 2000) and urea (Alonso-Sáez et al., 2012) as a carbon source. The metabolic predictions for these two orders point in the direction of mixotrophy. The predicted nitrogen fixation capability is also interesting. Given the fact that these two orders were mainly observed in oligotrophic water bodies, carbon and nitrogen fixation would be advantageous capabilities.

### Acidobacteria

#### Phylogeny and taxonomy

**Figure 5:**
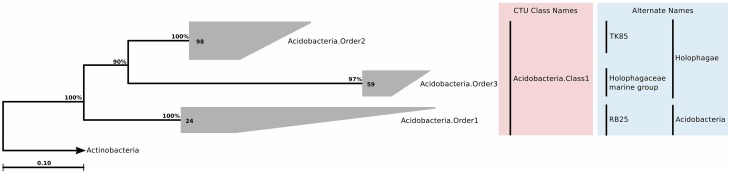
**Phylogenetic trees illustrating the relationships among SSU rRNA gene sequences belonging to uncultured marine *Acidobacteria***. Other details of the figure are same as Figure 1.

Despite the overall low number of isolated *Acidobacteria*, 16S rRNA genes of this phylum have been detected in very different ecosystems, as for example soil habitats (Kuske et al., 1997; Dunbar et al., 2000), freshwater habitats (Briée et al., 2007), hotsprings (Barns et al., 1999), and marine sponges (Hentschel et al., 2006). Currently, the phylum is subdivided into 26 subgroups or subdivisions (Barns et al., 2007), reflecting its phylogenetic depth, which is comparable to *Proteobacteria* or *Firmicutes*. Within this phylum we found 181 sequences that were of marine water column origin. In the original SILVA guide tree, these sequences were associated with classes *Holophagae* and *Acidobacteria*, but remained unclassified at deeper levels. Upon tree reconstruction and CTU application, the sequences constituted a single class, which contained three different orders, three families, and seven genera (Figure 5; Table S2).

#### Habitat

Acidobacterial 16S rRNA gene sequences have been detected in the marine environments, including deep-sea plankton (DeLong et al., 2006). Their actual diversity, relative abundance, and ecological role in the oceans remain unknown. Recent studies on several bathypelagic zones from different ocean basins revealed a high percentage of *Acidobacteria*-related sequences, suggesting that members of this phylum could account for an important fraction of the microbial community in these waters (Brown et al., 2009; Eloe et al., 2011; Quaiser et al., 2011). Within the ICoMM samples, acidobacterial taxa were rare in all marine biomes, but showed a slight increase in relative abundance in the mesopelagic and bathypelagic zones of the oceans, consistent with other reports (Figure 2). Of the three orders, members of the *Acidobacteria*.Order1 showed highest relative abundance, and were detected across coastal and open ocean waters of varying productivity, in addition to anoxic water bodies (Figure 3). Consequently, the species association test, which tests whether the null ecological hypothesis that the relative frequency of the taxa of interest is not higher in the target site group than in other groups, showed that *Acidobacteria*.Order1 relative abundance was highest in low productivity ocean water (Table 1). In further support of the deep ocean waters preference of *Acidobacteria*.Order1, we detected a significant positive correlation with depth, and a negative correlation with temperature (Figure 4).

#### Metabolism predictions

Soil dwelling *Acidobacteria* are capable of nitrate and nitrite reduction (Ward et al., 2009), but key enzymes of these pathways were not predicted by PICRUSt for marine *Acidobacteria* (Table S5). It has been suggested that deep ocean *Acidobacteria* may be involved in the degradation of recalcitrant organic carbon sources (Quaiser et al., 2008). The plethora of glycosidases, involved in starch, cellulose, and chitin degradation predicted by PICRUSt support this role. Furthermore, we also found evidence for sulfatases, which may be used for efficient degradation of sulfated glycopolymers from marine aggregates, as has been described for *Planctomycetes* (Glöckner et al., 2003) All orders were capable of degrading peptidoglycan, peptides, and polyamines. As no nitrogen assimilation enzymes were predicted for any of the orders, these degradation enzymes could play a role in both organic nitrogen acquisition and as an energy source. Overall, the distributions, relations to oceanographic parameters and nutrients, as well as metabolic predictions suggest a heterotrophic lifestyle for marine *Acidobacteria*. The positive correlation with inorganic nutrients, and the negative correlation with chlorophyll *a* concentrations could as well be due to their increased abundance in deeper waters. It is likely that they are preferentially particle-associated.

### Actinobacteria

#### Phylogeny and taxonomy

**Figure 6:**
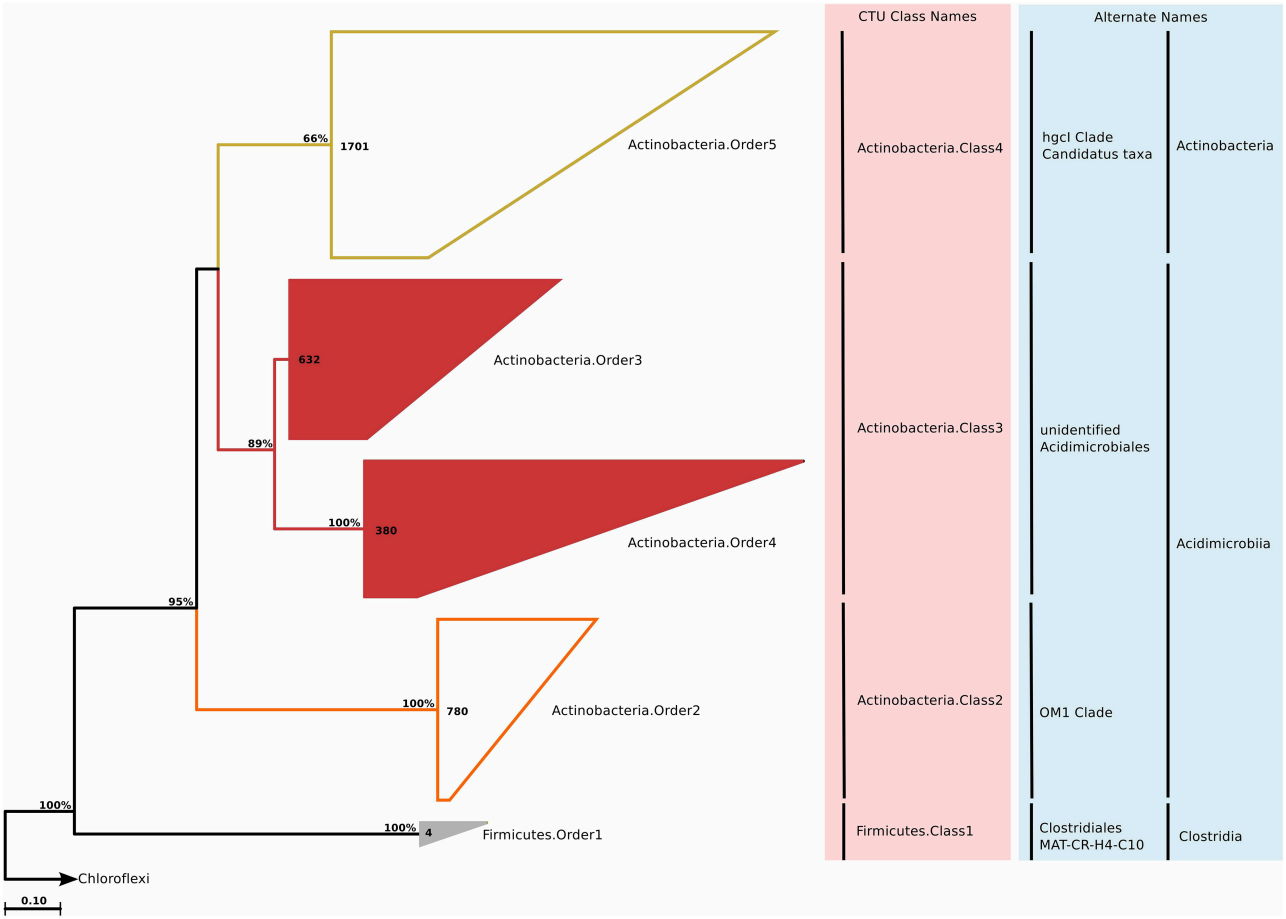
**Phylogenetic trees illustrating the relationships among SSU rRNA gene sequences belonging to uncultured marine *Actinobacteria* and *Firmicutes***. Other details of the figure are same as Figure 1.

*Actinobacteria* is one of the largest and most thoroughly studied phylum in the bacterial domain. It is also one of the most cultivate rich phyla, due to their importance in health, agriculture, and biotechnology. Cultivated marine *Actinobacteria* are mostly represented by *Actinomycetes*, but it has been suggested that the described actinobacterial diversity in the marine environment represents only a small subset of the existing diversity (Jensen and Lauro, 2008) In our study, we identified approximately 3500 sequences of *Actinobacteria* designated to be included in marine water column clades. After phylogenetic reconstruction and CTU application, these sequences were distributed across three classes and four orders; of these the *Actinobacteria*.Order3 and *Actinobacteria*.Order4 were previously not described (Figure 6). Both of these orders were quite diverse and split up into 14 genera (Table S2).

#### Habitat

The marine *Actinobacteria* group was first reported by Fuhrman et al. (1993) in their study of 16S rRNA clone sequences from the coast of Southern California and Bermuda. Currently, they are recognized as ubiquitous members of the marine bacterioplankton (Giovannoni and Stingl, 2005), and can constitute up to 10% of a bacterioplankton community (Yilmaz et al., 2012). Most of the actinobacterial observations can be attributed to the OM1 group, which is known to be most abundant near the deep chlorophyll maximum. The acI clade is a freshwater clade, but it is known to also occur in estuarine and coastal waters. The new actinobacterial clades seemed to be most dominant in the mesopelagic zones, although their relative abundances in the bathypelagial and abyssopelagial were also considerable (Figure 2). Partitioning of the ICoMM samples by water body and productivity revealed that *Actinobacteria.*Order4 is probably not truly an oceanic bacterioplankton, as the relative abundance of this clade peaked in various brackish water environments (Figure 3). *Actinobacteria.*Order3 presented a different pattern: it was more prominent in anoxic basins such as the Cariaco or Black Sea, but also detectable at a relative abundance of up to 1.5% in ocean waters. Within anoxic water bodies, *Actinobacteria.*Order3 had highest relative abundance in the mid-productivity range (Figure 3). Previous reports have indeed found *Actinobacteria* in anoxic basins, as well as in the meso- and bathypelagic zones of the oceans, possibly being particle-attached (Eloe et al., 2011; Wright et al., 2012; Rodriguez-Mora et al., 2013). This is in contrast to the lifestyle suggested by Ghai et al. for the OM1 clade *Actinobacteria* (Ghai et al., 2013), in which they report a preferential abundance of *Candidatus Actinomarina* in the photic zone. Therefore, we suggest that the *Actinobacteria.*Order3 of *Actinobacteria* should be related to either those reported in anoxic basins or meso- and bathypelagic zones.

#### Metabolism predictions

A large number of studies on marine *Actinobacteria* focused solely on secondary metabolite production (Bull and Stach, 2007), therefore the metabolism of marine *Actinobacteria* is still largely unknown. However, a recent genome reconstruction by Ghai et al. suggests a photoheterotrophic and aerobic lifestyle, and a capability to take up phosphate and phosphonate (Ghai et al., 2013). The PICRUSt predictions (Table S6) for the orders we investigated suggested a potential for cellulose, hemicellulose, and chitin degradation in terms of carbon metabolism. Interestingly, a cyanophycinase enzyme was also predicted, indicating that these orders may be taking advantage of nitrogen-rich cyanophycin.

### Bacteroidetes

#### Phylogeny and taxonomy

**Figure 7:**
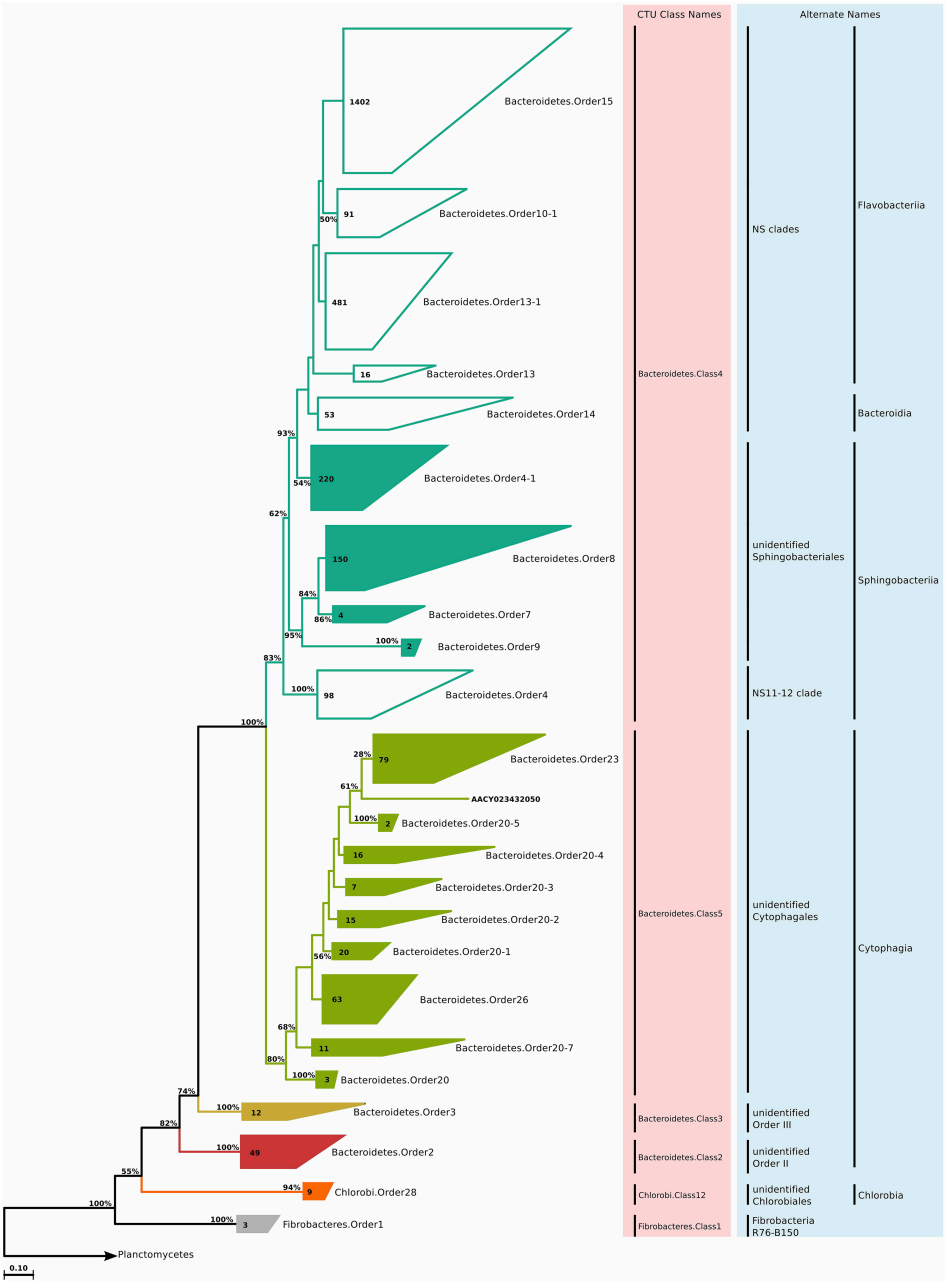
**Phylogenetic trees illustrating the relationships among SSU rRNA gene sequences belonging to uncultured marine *Bacteroidetes, Chlorobi*, and *Fibrobacteres***. Other details of the figure are same as Figure 1.

The phylum *Bacteroidetes*, especially the classes *Sphingobacteriia, Flavobacteriia*, and *Cytophagia*, constitute a big portion of the classically culturable marine bacterioplankton. Despite successful cultivation of many representatives, the relatively large diversity of this phylum still results in a large fraction of uncultured members. Furthermore, a data set by Alonso et al. (2007) indicated that cultivation-independent techniques and isolation approaches have recovered almost equally sized and virtually non-overlapping fractions of the currently known diversity within this phylum, making this phylum of further interest. The phylogenetic reconstruction and CTU application for approximately 3000 environmental clone sequences revealed three classes—*Bacteroidetes*.Class2 and *Bacteroidetes*.Class3 representing some *Cytophagia, Bacteroidetes*.Class4 representing sequences previously assigned to *Bacteroidia, Sphingobacteriia*, and *Flavobacteriia*, and finally *Bacteroidetes*.Class5 representing another group of sequences previously assigned *Cytophagia* (Figure 7). The new phylogenetic reconstruction grouped sequences of previously distinct classes, and separated sequences that were previously grouped in the same class. It is important to note that despite the recent taxonomic reorganization, the *Bacteroidetes* phylum still contains many polyphyletic groups and obvious taxonomic misassignments. Therefore, our findings are plausible as both a phylogenetic and sequence identity component was used for taxa delineation.

#### Habitat

Members of the phylum *Bacteroidetes* are found in a variety of marine environments including coastal and offshore waters (Eilers et al., 2001; Kirchman et al., 2003; DeLong et al., 2006), sediments (Musat et al., 2006), hydrothermal vents (Kormas et al., 2006) and polar regions (Brinkmeyer et al., 2003). Further, they are common both as free-living bacterioplankton and as attached to organic aggregates (DeLong et al., 1993; Eilers et al., 2001), and they can be associated with marine animals or phytoplankton (Grossart et al., 2005; Webster and Taylor, 2012). Generally speaking, *Bacteroidetes* often constitute the most abundant group of bacteria in coastal pelagic habitats, and they degrade complex and polymeric organic matter of different origins, rather than monomers (González et al., 2008).

Among the ICoMM samples, the relative abundances across different depths of neritic and oceanic regions were almost equal. These results could indicate that the new clades found in *Bacteroidetes* may represent order-level taxa that prevail in below the epipelagic zone (Figure 2). Relative abundances of these clades in anoxic waters were generally low or close to zero, as opposed to previous phyla that were considered (Figure 3), and as opposed to previous studies of, for example, the Saanich Inlet (Zaikova et al., 2010) In fact, most orders appeared in highly productive coastal and oceanic waters. Specifically, *Bacteroidetes*.Order2, *Bacteroidetes*.Order9, *Bacteroidetes*.Order20-4, and *Bacteroidetes*.Order20-7 have significantly higher relative abundances in high productivity coastal waters (Table 1). The only order that was enriched in ocean waters was *Bacteroidetes*.Order26. Most orders were negatively correlated with depth, except *Bacteroidetes*.Order26 and *Bacteroidetes*.Order4-1, which showed positive correlations (Figure 4). On the other hand, most orders were observed to positively correlate with temperature, except for *Bacteroidetes*.Order4-1 (Figure 4). Depth-specific phylotypes, but no phylogenetic clades, of *Bacteroidetes* have been reported previously (Diez-Vives et al., 2014). *Bacteroidetes*.Order4-1 and *Bacteroidetes*.Order26 could represent these yet unidentified phylogenetic clades of marine *Bacteroidetes*. The isolation sources of the full-length sequences for these clades also suggest a deep ocean niche, for example ambient hydrothermal vent water, or the Puerto Rico trench.

#### Metabolism predictions

In aquatic habitats, *Bacteroidetes* are abundant during and following algal blooms (Pinhassi et al., 2004), showing a preference for consuming polymers rather than monomers (Cottrell and Kirchman, 2000). In the oceans, the main lifestyle of *Bacteroidetes* is particle-attached and polymer-degrading. Therefore, their genomes are rich in proteases, glycosidases, and polysaccharide binding domains (Bauer et al., 2006; Gomez-Pereira et al., 2012; Fernandez-Gomez et al., 2013). For example, the genome of *Formosa agariphila* harbors 129 proteases and 88 glycosidases (Mann et al., 2013). PICRUSt predicted (Table S6) a number of proteases and glycosidases for all *Bacteroidetes* orders. Further, the orders associated with productive coastal waters had a greater variety of glycosidases compared to the single oceanic water order. Sulfatases and tonB transporter predictions also further supported that these orders would be degrading polymeric matter. All orders from *Bacteroidetes*.Order20-1 to *Bacteroidetes*.Order20-5 were predicted to have all enzymes for a complete denitrification pathway. Although all these orders were found primarily in the oxygenated waters, it is probable that if they become particle-associated, they might utilize denitrification within the microscale patches of these particles. Additionally, the prediction of RuBisCo for two orders, *Bacteroidetes*.Order20-4 and *Bacteroidetes*.Order20-7, indicated a potential for autotrophy. For two orders, *Bacteroidetes*.Order3 and *Bacteroidetes*.Order8, carbon monoxide dehydrogenase (CODH) was predicted. There are several available descriptions of aerobic marine CO oxidizers (King, 2003; Cunliffe, 2011), including members of the *Proteobacteria* and *Bacteroidetes*. Given that *Bacteroidetes*.Order3 was mostly found in low productivity waters, it could be utilizing aerobic CO oxidation to generate energy.

### Minor phyla—chlorobi, fibrobacteres, and firmicutes

#### Phylogeny and taxonomy

*Chlorobi* (Figure 7), *Fibrobacteres* (Figure 7), and *Firmicutes* (Figure 5) are not traditionally considered to be of marine origin, although we found small clades (nine, three, and four sequences, respectively) in our survey of clone sequences. *Chlorobi* clone sequences were not phylogenetically diverse, and constituted a single genus. *Fibrobacteres* sequences, on the other hand, were assigned to a single order and family, but each individual sequence represented a unique genus. Finally, *Firmicutes* sequences also formed a single genus (Table S2).

#### Habitat

The distribution of these phyla according to the ICoMM dataset did not show clear patterns (Figures 2, 3). No sequence tags were classified as *Chlorobi*, therefore we concluded that this clade is not considered a marine clade. *Fibrobacteres* was only identified in neritic surface waters, and it is uncertain whether this clade is an actual resident. The isolation sources of the full-length 16S rRNA sequences also did not provide more insights, since they were either animal-associated or coming from the Saanich inlet. Considering the presence in anoxic waters and worm/sponge hosts, it is possible that this clade represents chemoautotrophic, possibly symbiotic organisms. Similar to *Fibrobacteres*, it is also uncertain whether members of the *Firmicutes* clade are actual residents or allochthonous microbes. As some of the full-length sequences originated from hypersaline environments, and two unidentified sea samples, it is certain that the clade is salinity tolerant.

#### Metabolism predictions

PICRUSt predicted a capability for nitrogen and carbon fixation, and a very limited number of degradation enzymes for both *Fibrobacteres* and *Firmicutes*. These predictions support an anaerobic and chemolithoautotrophic lifestyle of these microorganisms (Table S6).

### Chloroflexi

#### Phylogeny and taxonomy

**Figure 8:**
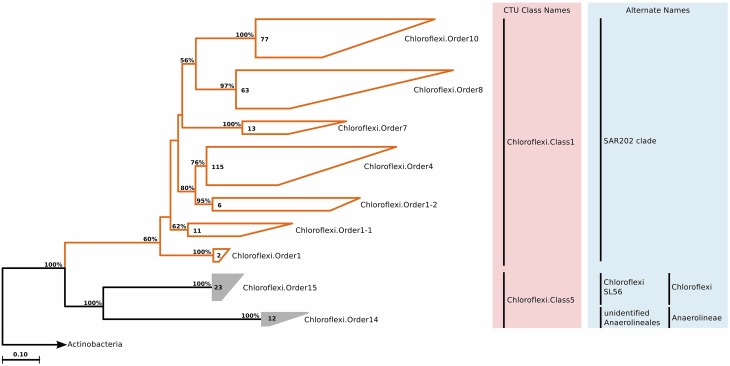
**Phylogenetic trees illustrating the relationships among SSU rRNA gene sequences belonging to uncultured marine *Chloroflexi***. Other details of the figure are same as Figure 1.

*Chloroflexi* is a phenotypically diverse phylum, encompassing aerobic thermophiles, anoxygenic phototrophs, and anaerobic chemolithotrophs. Relative to the current size of the phylum (around 9000 full-length 16S rRNA sequences), few isolates exist (21 named genera), and most of these isolates are found within the class *Anaerolineae*. The phylum is divided into six classes, however whether they all truly belong to the *Chloroflexi* is under debate (Gupta et al., 2013). The SAR202 cluster is a deep branch of *Chloroflexi*, and it is an extensively studied marine clade. According to our phylogenetic and CTU analyses, SAR202 shows extensive internal diversity, and can be separated into seven order-level groups, adding three more to the four subclusters suggested a decade ago (Morris et al., 2005). Further, we identified another 35 clone sequences that did not belong to the SAR202 clade, but still had marine origin. They formed a class-level clade within *Chloroflexi*, and contained two orders (Figure 8), with each order containing a single family and genus (Table S2).

#### Habitat

SAR202 was one of the first groups reported to show strong depth stratification. Further, it has also been repeatedly recovered from the deep sea (Brown et al., 2009). It has been shown that deeper waters can contain 66% more SAR202 cells relative to the surface, and that the cell counts of this clade reach a maximum just below the deep chlorophyll maximum (Morris et al., 2004). Distributions of the two orders exhibit two prominent environment types, anoxic for *Chloroflexi*.Order14 and brackish for *Chloroflexi*.Order15, which were also in accordance with the isolation sources of the clone sequences (Figure 3). No significant correlations were observed for *Chloroflexi*.Order14, whereas *Chloroflexi*.Order15 correlated positively with depth, chlorophyll *a*, and dissolved oxygen concentration, but negatively with salinity (Figure 4). The distribution data suggests that members of *Chloroflexi*.Order15 are bacterioplankton primarily adapted to brackish-marine transition zones, while *Chloroflexi*.Order14 is primarily composed of bacterioplankton adapted to life in anoxic marine waters. It is conceivable that *Chloroflexi*.Order14 represents the OTU detected by Rodriguez-Mora and colleagues, in their study of the Cariaco basin bacterioplankton diversity patterns (Rodriguez-Mora et al., 2013).

#### Metabolism predictions

According to PICRUSt predictions, *Chloroflexi*.Order14 does not harbor key enzymes for degradation of organic compounds. This is in contrast to previous assumptions of an adaptation of *Chloroflexi* to scarce food availability in the deep oceans, for which we would expect a more diverse degradation machinery to utilize recalcitrant organic compounds and other more labile sources such as sinking organic material (Cho and Azam, 1988; Jørgensen and Boetius, 2007). Therefore, we suggest that this order does not necessarily follow a heterotrophic lifestyle, but rather is capable of a range of metabolic processes, including denitrification and carbon fixation. Another interesting metabolism for *Chloroflexi*.Order14 would be acetogenesis, given that PICRUSt predicted both CODH and formyltetrahydrofolate synthetase (MTHFD), and that the order occupies anoxic water bodies.

### Deferribacteres/caldithrix

#### Phylogeny and taxonomy

**Figure 9:**
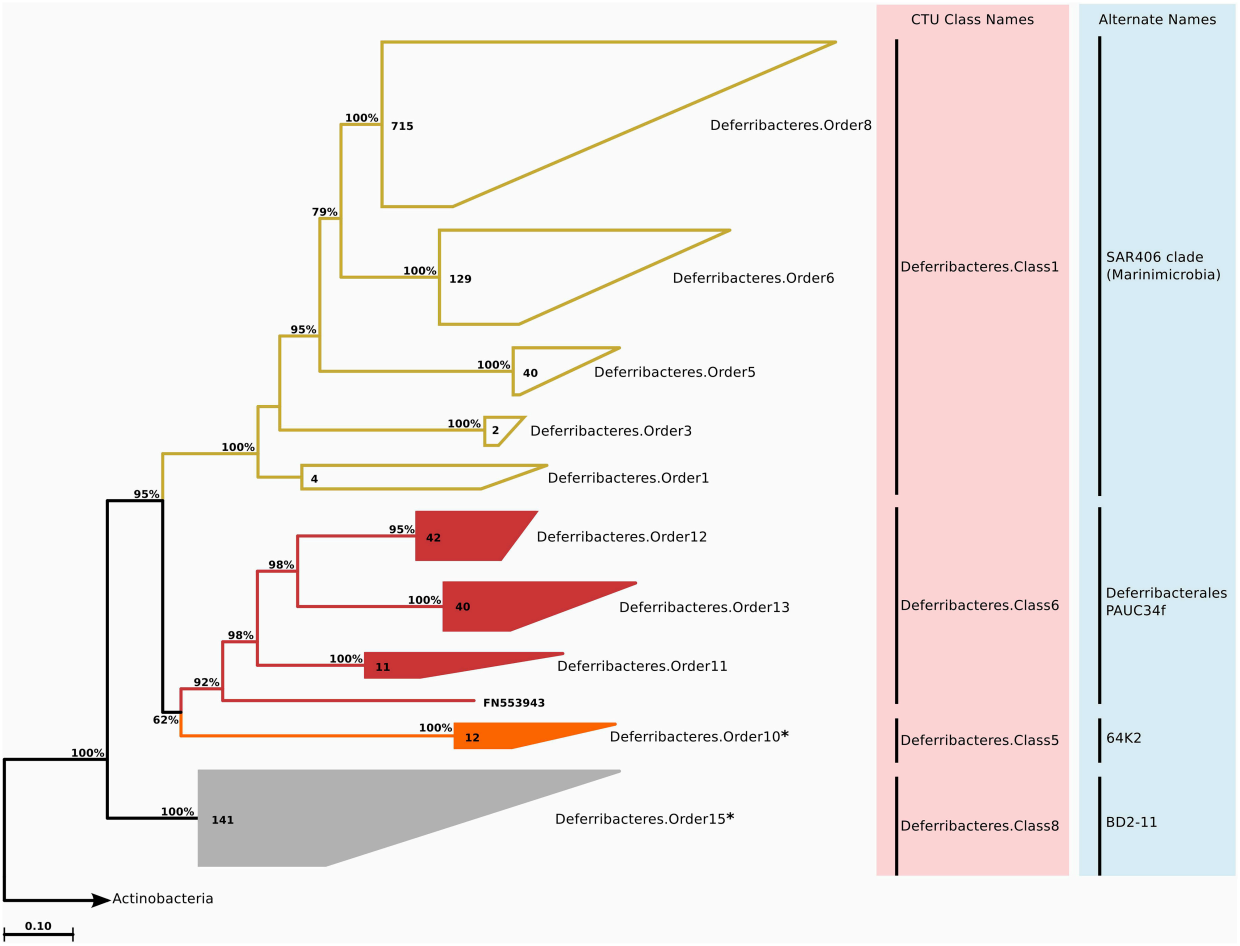
**Phylogenetic trees illustrating the relationships among SSU rRNA gene sequences belonging to uncultured *Deferribacteres/Caldithrix* group**. Other details of the figure are same as Figure 1. The groups Deferribacteres.Order10 and Deferribacteres.Order15 marked with an asterisk were previously associated with unidentified *Halanaerobiales* (*Clostridia*) and *Gemmatimonadetes* groups, but due to sequence identity values of above 75%, they were placed together with *Deferribacteres*/*Caldithrix*.

SAR406 (Marine Group A) appears as a major division of Bacteria, and has been recently named “*Marinimicrobia*” (Rinke et al., 2013). Before this single-cell genomics study, the only cultivated organism that was related to this group was *Caldithrix abyssi*, which is currently classified as a member of the *Deferribacteres* phylum in most nomenclatural and rRNA databases. This classification is possibly erroneous, as even the original description of *Caldithrix abyssi* does not suggest a strict association, and states that bootstrap support for placing *Caldithrix abyssi* together with other *Deferribacteres* members is weak (Miroshnichenko, 2003). Within the SAR406/*Caldithrix* clade, we identified several additional groups that were of marine origin (around 400 sequences), but not associated with either SAR406 or *Caldithrix* (Table S2). Phylogenetic reconstruction and CTU application revealed that in fact multiple classes exist within this group (Figure 9). The novel clades showed varying phylogenetic diversity. For instance, *Deferribacteres*.Order15 was one of the most diverse clades in this study, and based on sequence identity and tree topology, 21 genera were predicted (Table S2).

#### Habitat

In the marine environment SAR406 clade is ubiquitously distributed, though its relative abundance in the mesopelagic zone is about five times higher than in the surface ocean. Further, previous studies focusing on SAR406 diversity and distribution in the dark ocean indicated that it is most prevalent and diverse in oxygen minimum zones and permanently or seasonally stratified anoxic basins (Fuchs et al., 2005; Stevens and Ulloa, 2008; Schattenhofer et al., 2009). Lastly, it is also known that the group shows seasonal oscillations and that they are positively correlated with chlorophyll *a* concentration (Gordon and Giovannoni, 1996; Cram et al., 2014).

The depth distribution of novel *Deferribacteres* clades in the ICoMM samples were similar to that of SAR406 clade, with a peak at and below the mesopelagic zone (Figure 2). An analysis of distribution across different water bodies revealed that Order15 had highest relative abundance in low productivity open oceans (Figure 3). Contrarily, *Deferribacteres*.Order11 was relatively more abundant in high productivity oceanic zones, while *Deferribacteres*.Order13 appeared to be more dominant in anoxic basins. *Deferribacteres*.Order10 and *Deferribacteres*.Order12 had low relative abundance (less than 0.01%), and were identified primarily in low productivity oceanic waters (Figure 3). Finally, in agreement with the distribution results, we observed that *Deferribacteres*.Order13 was negatively correlated to dissolved oxygen concentration (Figure 4). The results suggest that the new putative *Deferribacteres* orders preferentially live in oligotrophic oceans, but at and below the mesopelagic zone, hence the correlation to nutrients. While *Deferribacteres*.Order10, *Deferribacteres*.Order11, *Deferribacteres*.Order12, and *Deferribacteres*.Order15 would be more likely to inhabit the oxygenated water column, *Deferribacteres*.Order13 is more likely to inhabit anoxic water columns. In fact these distribution patterns are supported by another study, based on a fosmid carrying a 16S rRNA sequence related to SAR406 and *Deferribacteres*, which has shown adaptations to suboxic and dysoxic conditions (Wright et al., 2014).

#### Metabolism predictions

PICRUSt predicted (Table S6) the potential for harboring nifH genes for all *Deferribacteres* orders. A nitrogen-fixing nifU domain-containing protein is predicted for the *Caldithrix abyssi* genome, but not for any of the SAR406 contigs. Previous studies have shown that nifH genes can be amplified from non-cyanobacterial bacterioplankton (Bird et al., 2005), and it was also shown that these nifH genes were expressed in the mesopelagic zone (Hewson et al., 2007). Further, nitrogen fixation in oxygen minimum zones was demonstrated (Fernandez et al., 2011). Therefore, it is possible that these *Deferribacteres* to have nitrogen fixing ability, which would be advantageous in oligotrophic environments.

### Lentisphaerae

#### Phylogeny and taxonomy

**Figure 10:**
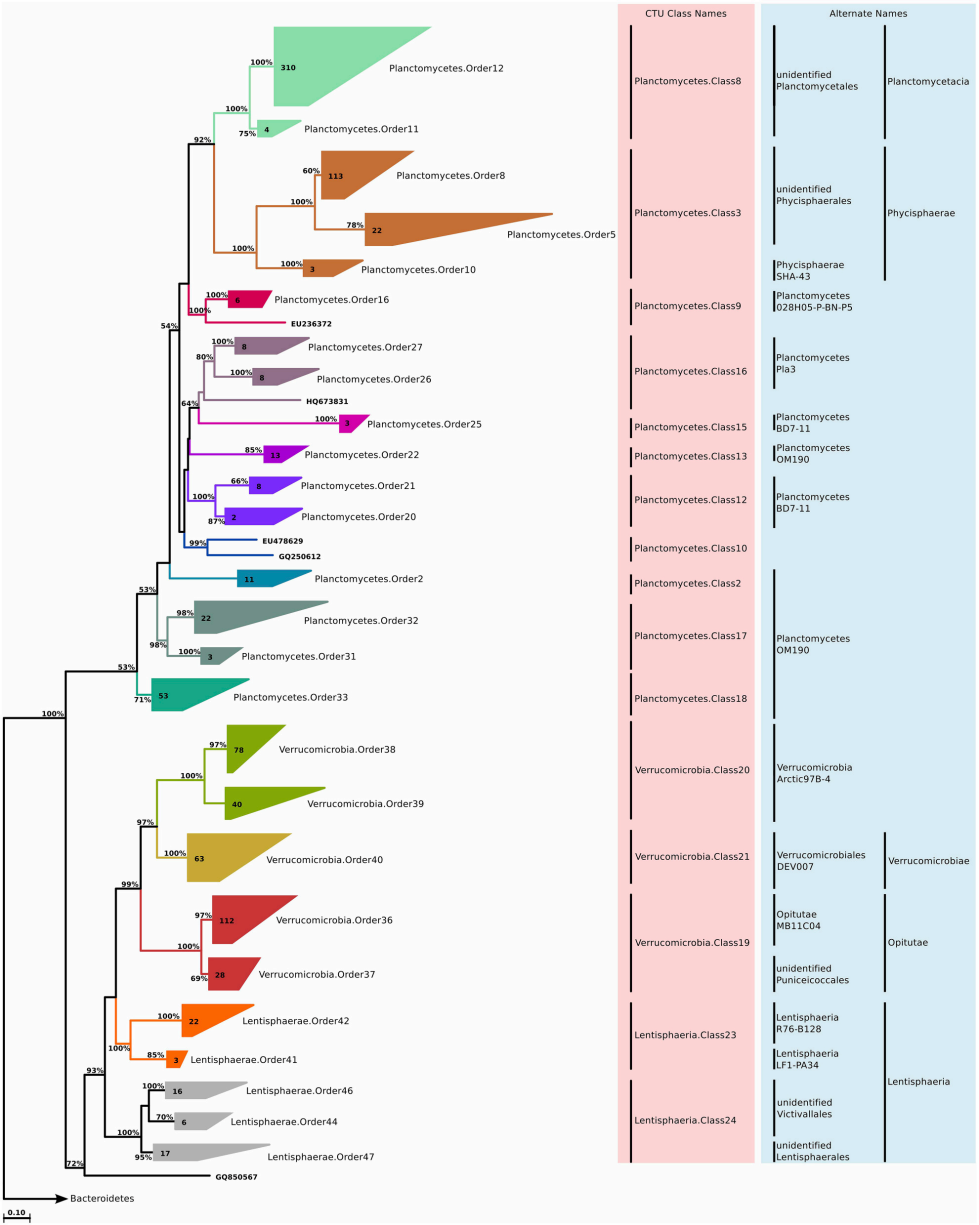
**Phylogenetic trees illustrating the relationships among SSU rRNA gene sequences belonging to uncultured marine *Planctomycetes, Verrucomicrobia*, and *Lentisphaerae***. Other details of the figure are same as Figure 1.

*Lentisphaerae* is a relatively small phylum (1200 sequences in SILVA Ref 111) that is closely related to *Planctomycetes, Verrucomicrobia*, and *Chlamydiae*. It currently encompasses two classes, *Lentisphaeria* and *Oligosphaeria*, and only four cultivated species. The 64 marine *Lentisphaeria* sequences were split into two classes, each affiliated to a distinct phylum (Figure 10). This observation points to the possibility of merging Lentisphaerae and Verrucomicrobia into one phylum, which would be supported by monophyly and sequence identity values above 75%. CTU methodology suggested five different orders, and further six families. The phylum overall was not very diverse, according to the available sequence data, and most of these families contained only a single genus (Table S2).

#### Habitat

Data on *Lentisphaerae* diversity, distribution, and abundance is rather scarce, but few studies have shown that they can be found in seawaters of the Arctic (Bano and Hollibaugh, 2002) and the Pacific Ocean (Cho et al., 2004), in deep-sea sediments (Li et al., 1999), and associated with marine animals (Alain et al., 2002). *Lentisphaera araneosa* is described as a common marine bacterium which occurs in relatively low numbers, accounting for less than 1% of total bacterial 16S rRNA (Cho et al., 2004).

In our results, *Lentisphaerae* was present across all biomes, but in low relative abundances. A small increase in relative abundances was observed below the mesopelagic zone (Figure 2). Across different water bodies, the orders seemed to be relatively more abundant in anoxic waters (Figure 3), however species association tests showed that *Lentisphaerae*.Order44 and *Lentisphaerae*.Order47 relative abundances were significantly higher in high productivity ocean waters, while *Lentisphaerae*.Order41 relative abundance was highest in the mid productivity ocean waters (Table 1). *Lentisphaerae*.Order42 and *Lentisphaerae*.Order46 were associated with anoxic waters, but this association was not significant. In support of the abundance of these orders in anoxic water bodies, negative correlations with dissolved oxygen concentration were also observed (Figure 4). These *Lentisphaerae* orders mostly inhabit productive water masses, but they are also found in anoxic water bodies. If they are also capable of producing transparent exopolymeric particles like *Lentisphaerae arenosa* (Cho et al., 2004), they might play a role in dissolved organic carbon production and carbon recycling in these environments.

#### Metabolism predictions

PICRUSt predictions (Table S6) suggested nitrogen fixation ability for *Lentisphaerae*.Order41 and *Lentisphaerae*.Order42. For *Lentisphaerae*.Order42, which was negatively correlated to nitrate concentrations, it would be an advantage to fix nitrogen. *Lentisphaerae*.Order47 is potentially capable of nitrate reduction, which would be in accordance with its positive correlation to nitrate concentration. With respect to sulfur metabolism, *Lentisphaerae*.Order41 and *Lentisphaerae*.Order42 were predicted to harbor Adenosine-5′-phosphosulfate reductase (aprA). aprA primarily acts in the reductive direction, but the oxidative-type is observed in sulfur oxidizing Bacteria (Meyer and Kuever, 2007). Although either of these activities have not been reported for *Lentisphaerae* species yet, the proposed habitat preferences of oxic-anoxic water interfaces suggests the sulfate reduction or nitrogen fixation capabilities, as has been reported for other benthic and biofilm forming bacteria (Bertics et al., 2013; Desai et al., 2013). In terms of nutrient acquisition, both sulfatases and glycosidases were predicted, but the sulfatases were restricted to orders from *Lentisphaerae*.Order44 to *Lentisphaerae*.Order47. This indicated that they are capable of degrading complex organic matter, which especially fits the distribution pattern observed for Order44, being most abundant in productive ocean waters.

### Planctomycetes

#### Phylogeny and taxonomy

*Planctomycetes* is a deeply branching phylum of Bacteria with several unique characteristics distinguishing it from all other bacterial groups, such as a cytoplasm divided into compartments by membranes, reproduction by budding, and a cell envelope that lacks peptidoglycan (Fuerst, 2005). Briefly, the phylum can be divided into the cultured portion, which includes *Planctomycetacia* and *Phycisphaerae* classes, and the uncultured portion, which includes the anammox bacteria (“*Brocadiales*”). The phylum can be considered mostly aquatic; with isolation sources that include brackish, freshwater and marine environments.

We identified clades that were associated with both *Planctomycetacia* and *Phycisphaerae* (Figure 10). The diversity within the phylum was large, and CTU method application suggested that for the given sequences, the phylum should be divided into at least 10 classes, 16 orders, and 43 families (Table S2). This result also suggests that the formal classes of *Planctomycetes* are in fact multi-class assemblages, and a reorganization of the phylum in light of the CTU methodology should be considered.

#### Habitat

In terms of abundance, *Planctomycetes* do not belong to the major players in the marine water column. They generally constitute less than 5% of the bacterioplankton community in coastal waters, and less than 1% in the open ocean (Rusch et al., 2007; Schattenhofer et al., 2009; Yilmaz et al., 2012). In the marine water column, they are known to colonize marine snow (DeLong et al., 1993; Rath et al., 1998; Crump et al., 1999), and their abundance increases in response to blooms in coastal waters (Morris et al., 2006).

We observed that *Planctomycetes* were relatively more abundant in the neritic mesopelagic zone, as well as in the oceanic mesopelagic zone and below (Figure 2). Across different water masses, the emerging pattern was higher relative abundances in anoxic water masses, as well as in high productivity coastal and oceanic waters (Figure 3). Specifically, *Planctomycetes*.Order16, *Planctomycetes*.Order22, and *Planctomycetes*.Order27 showed a significantly higher relative abundance in mid productivity ocean waters (Table 1). Two interesting groups were *Planctomycetes*.Order12 and *Planctomycetes*.Order33, which were in fact present across all water massed considered here. The correlations with physical and chemical parameters were almost uniform across all the orders. Specifically, depth and nutrient concentrations were positively correlated to the relative abundance, while temperature, dissolved oxygen and chlorophyll *a* concentrations were negatively correlated with relative abundance (Figure 4). Overall, it appears that marine *Planctomycetes* are mostly found in high productivity open ocean waters. Furthermore, they appear to be relatively more abundant in the mesopelagic zone rather than the surface waters, which would explain the correlation with depth and temperature. Despite the increased abundance of *Planctomycetes* in higher productivity areas, the negative correlation with chlorophyll *a* concentration is interesting. One possible explanation would be that this is an artifact of the higher relative abundance found in anoxic water bodies, which did not have such high chlorophyll *a* concentrations. Finally, we can conclude that these *Planctomycetes* orders are part of anoxic basin bacterioplankton communities. This would be in addition to other reports, where marine anoxic basin *Planctomycetes* are mainly anammox *Planctomycetes*, while the orders considered here belong to other clades (Fuerst and Sagulenko, 2011; Wright et al., 2012).

#### Metabolism predictions

In terms of functional capabilities, PICRUSt predicted (Table S5) the presence of dissimilatory nitrate reductase and aprA, for all orders except *Planctomycetes*.Order12. *Planctomycetes*.Order12 displayed a different functional prediction pattern for other metabolic capabilities, characterized by more sulfatases and glycosidases, as well as storage compound synthesis and phosphate acquisition genes. It is possible that *Planctomycetes*.Order12 represents a marine *Planctomycetes* group that is found in association with marine snow particles, with a heterotrophic lifestyle. Finally, all *Planctomycetes* orders were predicted to contain CODH, possibly acting as an additional source of energy and carbon.

### Proteobacteria

#### Phylogeny and taxonomy

*Proteobacteria* is one of the most extensively studied, and incidentally also the most sequence-rich phylum in the bacterial domain. Many proteobacterial cultivates from the marine environment exist, but these classically cultivable bacteria generally represent eutrophic species that would be considered r-strategists or bloomers. Yet, countless uncultivated clades have been identified since early 1990s, including the ubiquitous SAR11 clade, gammaproteobacterial OMG clades, and *Deltaproteobacteria* SAR324 (Marine Group B) clade. With more recent cultivation and single-cell genomics approaches, many of these clades have been brought into culture, or have had their genomes sequenced (Giovannoni et al., 2005; Grote et al., 2011; Swan et al., 2011; Huggett et al., 2012).

**Figure 11:**
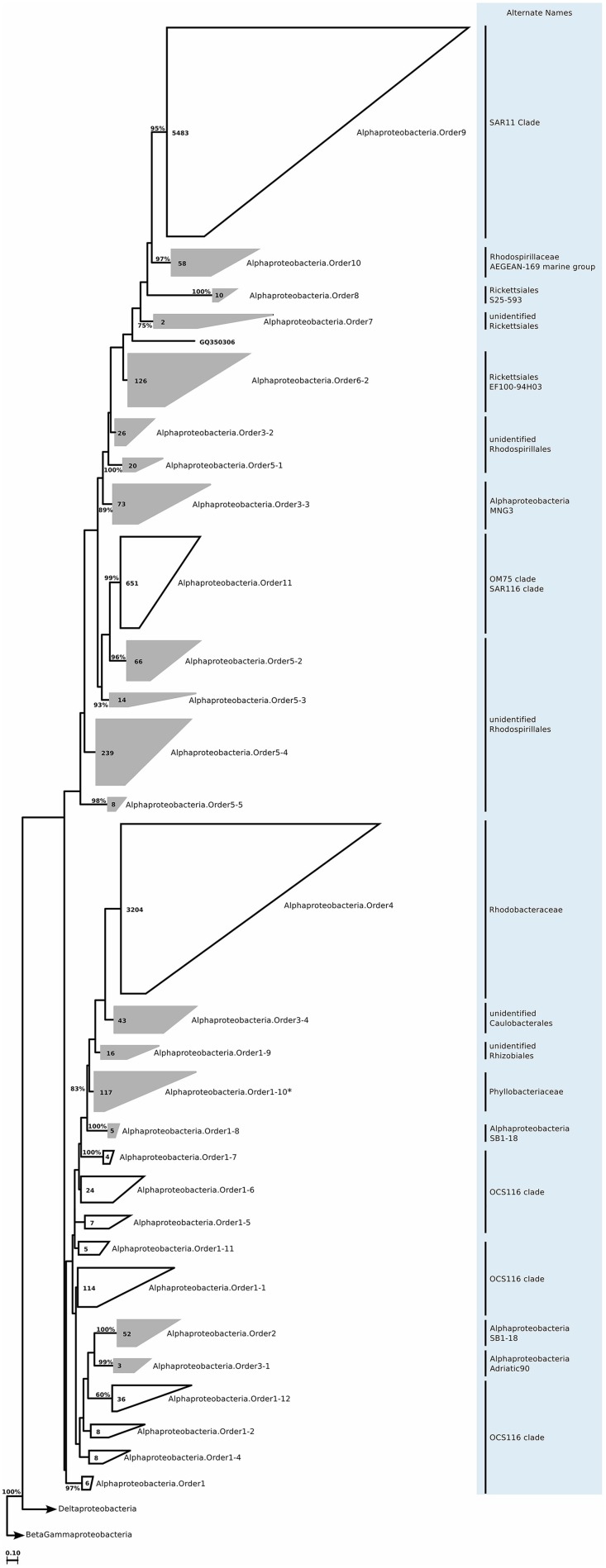
**Phylogenetic trees illustrating the relationships among SSU rRNA gene sequences belonging to uncultured marine *Alphaproteobacteria***. Other details of the figure are same as Figure 1. *Alphaproteobacteria*.Order1-10 marked with an asterisk was not included further analysis, as it was found to contain *Nitratireductor indicus* sequences in a more recent SILVA Ref dataset release.

**Figure 12:**
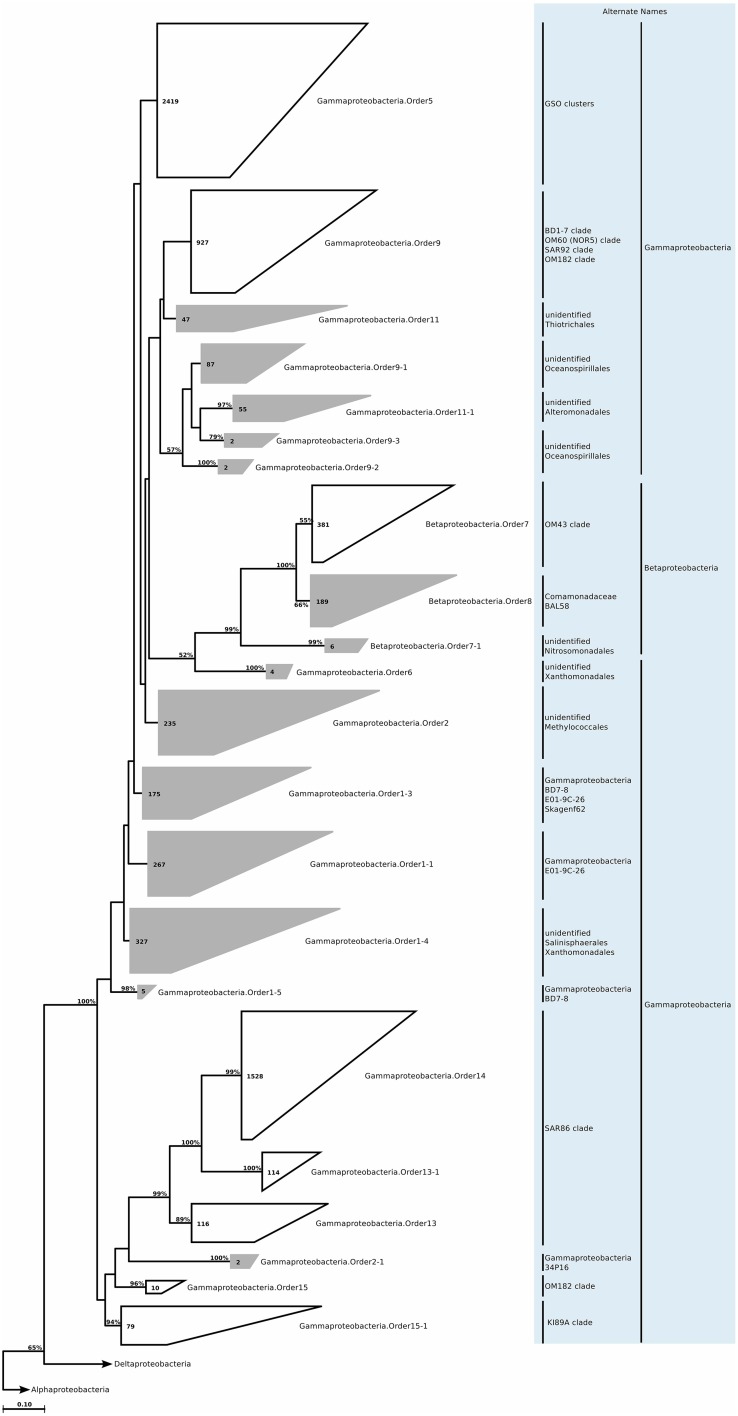
**Phylogenetic trees illustrating the relationships among SSU rRNA gene sequences belonging to uncultured marine *Beta- and Gammaproteobacteria***. Other details of the figure are same as Figure 1.

**Figure 13:**
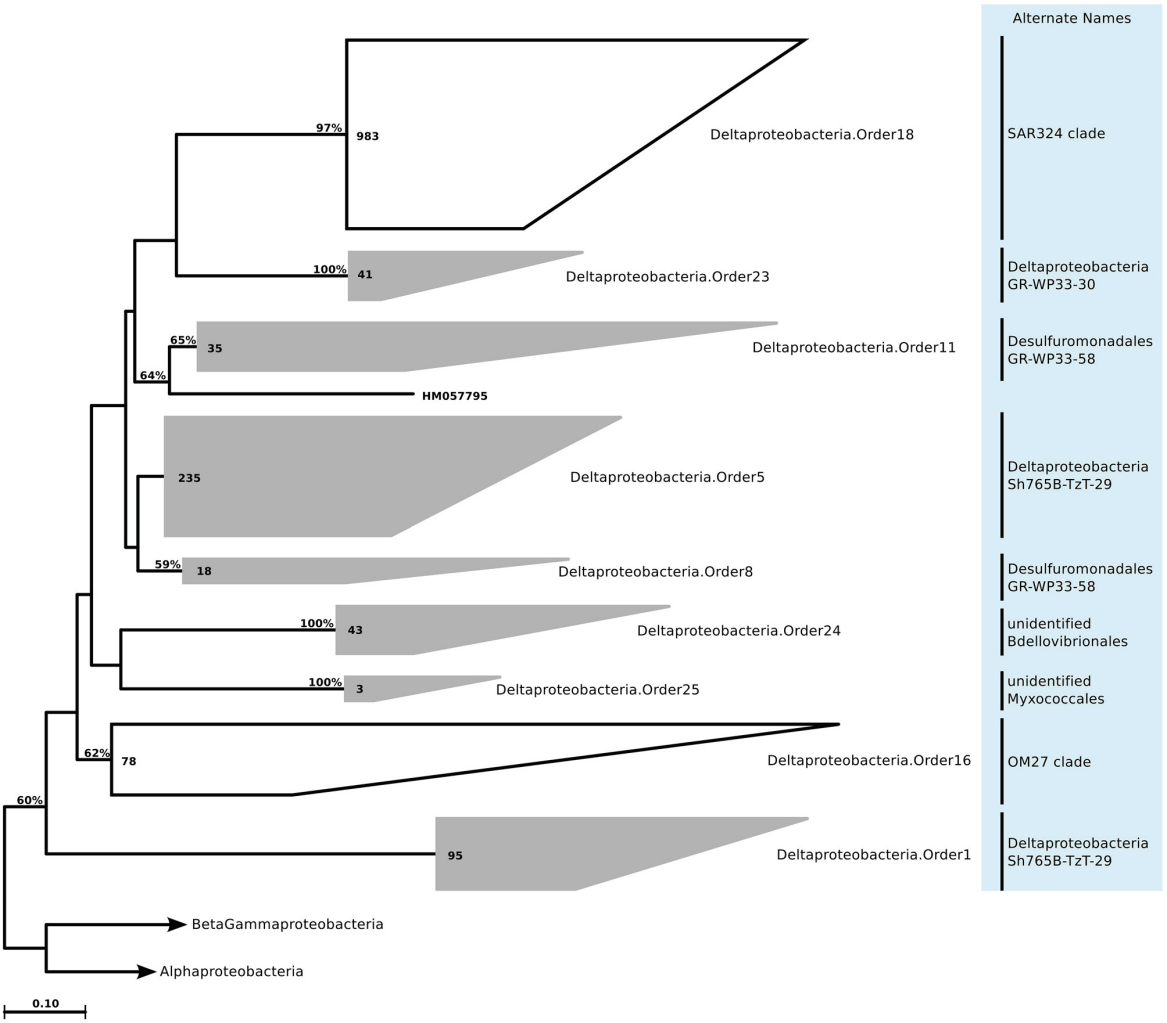
**Phylogenetic trees illustrating the relationships among SSU rRNA gene sequences belonging to uncultured marine Deltaproteobacteria**. Other details of the figure are same as Figure 1.

We still found around 30 order level clades, which had no association to previously known marine proteobacterial clades. For instance among the 10,500 *Alphaprotebacteria* sequences that we identified as marine origin, 741 were found in clades that could not be associated to known clades. These sequences were split into 13 orders, and further 29 families, a number comparable to the 15 known clades (Table S2; Figure 11). We reconstructed the trees for *Beta-* and *Gammaproteobacteria* together, as they behave as a single phylogenetic unit with strong taxonomic significance. In our tree reconstruction, *Betaproteobacteria* appeared as a monophyletic clade, embedded between two other *Gammaproteobacteria* clades (Figure 12). In this case, half of the 7000 marine origin sequences were not associated to the known clades. Together, they constituted 15 orders and 36 families. The diversity within these groups was rather large, with up to 15 genera annotated within one of the gammaproteobacterial families (Table S2). Finally, within *Deltaproteobacteria*, we identified 1500 sequences of marine origin, and out of this number 470 were not associated to clades OM27 and SAR324 (Table S2; Figure 13). These sequences were annotated as four distinct orders, which were then further annotated as 14 families. The diversity was again rather high, for example *Deltaproteobacteria*.Family14 contained 17 distinct genera (Table S2).

### Alphaproteobacteria

#### Habitat

*Alphaproteobacteria* is probably the most well-known and most abundant of all marine *Proteobacteria*. The SAR11 clade can constitute up to 33% of many euphotic zone communities and 25% of mesopelagic communities (Morris et al., 2002). It was previously considered an independent order of *Alphaproteobacteria*, though the sequence divergence within the group indicates that it is in fact a multi-class group (Yarza et al., 2014). Regardless of what the real taxonomic rank of SAR11 clade is, the group has been divided into subgroups, and then even further into phylotypes. These subgroups and phylotypes have since been found to be enriched differentially in open ocean or coastal samples (Schwalbach et al., 2010; Brown et al., 2012). In the surface ocean, the most frequent are subgroups 1a (contains *Candidatus Pelagibacter ubique*), 1b, and 2 (Morris et al., 2002; Carlson et al., 2009). On the contrary, subgroup 3 is more frequently found in coastal or brackish water samples (Brown et al., 2012). The lifestyle strategy of SAR11 is that of a K-strategist (slow growing and relatively dominant); in order to deal with oligotrophic conditions, the free-living organisms have streamlined their metabolisms and genomes, and thereby decrease their nutrient requirements (Giovannoni et al., 2005; Zhao and Andersson, 2014). Another clade that is presumably ecologically and metabolically similar to SAR11 is the SAR116 clade. The group is associated with *Rickettsiales*, and contains one cultured representative, *Candidatus Puniceispirillum marinum*. They are most frequently found in the euphotic zone, and they bloom during summer stratification (Treusch et al., 2009). Similar to SAR11, their abundance is negatively correlated to bacterial abundance and chlorophyll *a* concentration (Mayali et al., 2010). In addition to SAR11 and SAR116, yet another K-strategist is the OM75 clade (Rappe et al., 1997); a genus level clade presumably associated with *Rhodospirillaceae*. As no cultured representatives, or genomic data exists for the clade, the free-living oligotrophic lifestyle is currently a speculation (Nelson et al., 2014). Another alphaproteobacterial group that is generally considered having a different ecological niche than the SAR11, SAR116, and OM75 clades is the uncultured OCS116 clade (Suzuki et al., 1997). In oligotrophic environments, they bloom during spring, but they also persist during the entire year in the deep chlorophyll maximum (Treusch et al., 2009; Morris et al., 2012). In fact it has been suggested that this clade could have a meso-oligotrophic lifestyle (Nelson et al., 2014). The *Roseobacter* lineage completes the range of marine alphaproteobacterial lifestyles: copiotrophs that are metabolically versatile and capable of rapid growth (Azam and Malfatti, 2007; Newton et al., 2010; Brown et al., 2014), although more recent studies have also shown genome streamlining similar to the SAR11 clade (Luo and Moran, 2014; Voget et al., 2015) They can constitute up to 20% of coastal water bacterioplankton communities, and up to 15% of open ocean communities (Buchan et al., 2005).

ICoMM samples analysis revealed that *Alphaproteobacteria* is still the most abundant among the newly recognized clades (Figure 2). These clades had a higher relative abundance below 200 m, as opposed to the expectation that marine *Alphaproteobacteria* would be more abundant in the epipelagic zone. Across different water body types, *Alphaproteobacteria*.Order10 was more abundant than the others, particularly in low productivity oceanic waters. *Alphaproteobacteria*.Order10 was followed by *Alphaproteobacteria*.Order5-4, which was found across all water body types, and had a peak at anoxic water. The rest of the orders had lower relative abundances, and they were present mostly in low to mid- productivity coastal and oceanic waters, in a manner similar to SAR11, SAR116, and OM75 clades (Figure 3). In terms of correlation to physicochemical parameters, the significant correlations we observed were with depth (negative except for *Alphaproteobacteria*.Order5-1), chlorophyll *a* (negative except for *Alphaproteobacteria*.Order5-1), temperature (positive for *Alphaproteobacteria*.Order6-2, *Alphaproteobacteria.*Order7, and *Alphaproteobacteria*.Order10), dissolved oxygen (negative), and nitrate/phosphate (positive for *Alphaproteobacteria*.Order5-4 and *Alphaproteobacteria*.Order5-5) (Figure 4). Taken together with the distribution data, these correlations also point toward a more oligotrophic lifestyle for these new marine alphaproteobacterial clades. We hypothesize that they occupy low productivity areas and respond to nutrient input.

#### Metabolism predictions

PICRUSt predicted (Table S6) the potential for incomplete dissimilatory nitrate reduction and denitrification pathways in orders from *Alphaproteobacteria*.Order3-1 to *Alphaproteobacteria*.Order3-4, as well as from *Alphaproteobacteria*.Order5-1 to *Alphaproteobacteria*.Order5-3. These orders were relatively abundant in anoxic waters, as well as in productive coastal waters where suboxic conditions can be found. Therefore, either process is plausible. Except for *Alphaproteobacteria*.Order3-4, no assimilatory nitrogen reduction enzymes were predicted. Possibly to counteract this, all orders contained aminopeptidases, oligopeptidases, and amidases. Additionally, nifH was predicted for *Alphaproteobacteria*.Order2, *Alphaproteobacteria*.Order5-2, *Alphaproteobacteria*.Order5-4, *Alphaproteobacteria*.Order5-5, and *Alphaproteobacteria*.Order6-2. It is possible to suggest that these orders could represent the previously observed heterotrophic nitrogen fixers (Halm et al., 2012). Given that *Alphaproteobacteria*.Order5-2 to *Alphaproteobacteria*.Order5-5 are related to *Rhodospirillales*, nitrogen and carbon fixation are likely metabolic capabilities (Dubbs and Tabita, 2004; Baldani et al., 2014). However, relatives of *Alphaproteobacteria*.Order2 and *Alphaproteobacteria*.Order6-2 are not known to contain such metabolic capabilities. CODH was predicted for almost all orders, excluding *Alphaproteobacteria*.Order3-4, *Alphaproteobacteria*.Order7, and *Alphaproteobacteria*.Order10, in accordance with previous reports, and possibly giving an additional means of energy conservation.

### Betaproteobacteria

#### Habitat

The *Betaproteobacteria* are a metabolically diverse group, and although they are common members of freshwater and soil communities, they are not as widespread or diverse in the marine environments compared to *Alpha*- or *Gammaproteobacteria*. A potentially significant and abundant coastal clade is OM43 (Rappe et al., 1997), which is related to obligate methylotrophs that use the ribulose monophosphate (RuMP) pathway for carbon assimilation and are unable to oxidize methane (Giovannoni et al., 2008; Huggett et al., 2012). Additionally, some *Betaproteobacteria* seem to be members of the rare biosphere, and bloom for short periods of time, for example during wintertime in the Arctic Ocean (Alonso-Sáez et al., 2014).

The new clades appeared to be not only present in the coastal biomes, but were also found in similar relative abundances across other oceanic biomes and depth zones (Figure 2). *Betaproteobacteria*.Order7-1 had low relative abundance (less than 0.2% at peak), and was mostly found in low productivity open ocean waters. *Betaproteobacteria*.Order8, on the other hand, had relative abundances of up to 5%, but this was restricted to brackish waters, and its relative abundance in marine waters was higher than that of *Betaproteobacteria*.Order7-1, with a tendency toward higher productivity (Figure 3; Table 1). The distribution pattern of *Betaproteobacteria*.Order8 were in accordance with the isolation sources of full-length sequences, which were mostly coastal sampling sites. However, since this order is mostly found in brackish waters, it is questionable if it is a native member of the marine water column.

#### Metabolism predictions

PICRUSt predictions (Table S6) suggested a potential for denitrification for all orders, dissimilatory sulfite reduction for *Betaproteobacteria*.Order8, and most interestingly, a methane monoxygenase in *Betaproteobacteria*.Order8. This order is related to *Burkholderiales*, which now includes a methylotrophic member (Nakatsu et al., 2006), and previous reports based on recovery of *Burkholderiales*-related 16S rRNA genes from methane seeps have suggested that *Betaproteobacteria* may conduct methanotrophy (Pernthaler et al., 2008). In the Baltic Sea, where the brackish water samples originate from, methanotrophy is well-documented (Berndmeyer et al., 2013), and it is possible that *Betaproteobacteria*.Order8 are members of this community. Lastly, PICRUSt also suggested carbon fixation via RuBisCo for both orders, and surprisingly low numbers of nutrient acquisition genes were predicted. For both orders, the predictions suggest a chemoautotrophic lifestyle.

### Deltaproteobacteria

#### Habitat

Within the class *Deltaproteobacteria*, two major physiologies dominate—the fruiting-body-forming *Myxococcales*; and anaerobic sulfate and sulfur reducing bacteria. Only two clades of marine water column *Deltaproteobacteria* are known. The first is the SAR324 (Marine Group B) clade, an independent branch within the class. This clade is primarily found in waters deeper than 500 m (Fuhrman and Davis, 1997; Wright et al., 1997), with little seasonal fluctuations (Treusch et al., 2009; Nelson et al., 2014). Recently, genomes of the SAR324 clade have become available. The findings underlined a flexible lifestyle, and suggested oxidation of carbon monoxide and methane, methylotrophy, and also motility and adhesion genes associated with a particle-attached lifestyle (Chitsaz et al., 2011; Swan et al., 2011). The other marine water column clade is OM27 (Rappe et al., 1997), associated with *Bdellovibrionales* according to the SILVA guide tree. Currently very little is known about this clade. It has been suggested that they are more frequent in lower latitudes (Yilmaz et al., 2012), and in these tropical waters they are mainly found in the epipelagic zone (DeLong et al., 2006; Pham et al., 2008).

According to our analysis of the ICoMM samples, the newly recognized clades were mostly associated with surface waters (Figure 2). *Deltaproteobacteria*.Order5 had the highest relative abundances, and it was present across all water body types, though its relative abundance was significantly higher in mid productivity open ocean waters (Figure 3; Table 1). The two other orders that had significantly higher relative abundances in a particular water body type were *Deltaproteobacteria*.Order1 and *Deltaproteobacteria*.Order23, found in high and low productivity open ocean waters, respectively. *Deltaproteobacteria*.Order8 was mostly found in anoxic waters, and although results were not significant (Figure 3; Table 1), as the order is related to *Desulfuromonadaceae*, anoxic waters would be a likely habitat.

#### Metabolism predictions

PICRUSt predicted (Table S6) the potential for nitrogen fixation for *Deltaproteobacteria*.Order5, *Deltaproteobacteria*.Order8, *Deltaproteobacteria*.Order11, and *Deltaproteobacteria*.Order23, as well as the potential to fix carbon via either 3-hydroxypropionate/malyl-CoA cycle or the reverse TCA cycle. Interestingly, several DNRA and denitrification genes were predicted for these orders as well, such as nitrite reductase (nrfA) and nitric oxide reductase (norB). In contrast to the other orders, *Deltaproteobacteria*.Order25 had a different set of predictions, characterized by organic nitrogen conversion to ammonia (glutamate dehydrogenase and urease), more nutrient acquisition genes (glycosyslases, peptidases, phosphateses, and iron transport systems). Given that this order is related to *Myxococcales*, and was relatively more abundant in high productivity coastal waters, it could be a heterotrophic clade that responds to nutrient input or phytoplankton blooms. Although not many predictions were made for *Deltaproteobacteria*.Order1, the fact that the majority of sequences for this order were recovered from high productivity oceanic waters, it could represent heterotrophs that respond to bloom situations. *Deltaproteobacteria*.Order5, *Deltaproteobacteria*.Order11, and *Deltaproteobacteria*.Order23 could represent mixotrophs given the variety of metabolic functions predicted for them, and finally *Deltaproteobacteria.*Order8 is possibly a sulfate reducing clade, capable of carbon fixation, since they are often found in the anoxic water column and possess sulfite reductases (dsrAB), as well as alternative carbon fixation pathways (Neretin et al., 2007).

### Gammaproteobacteria

#### Habitat

Several orders within *Gammaproteobacteria* contain almost entirely marine-origin species, such as *Oceanospirillales, Alteromonadales*, or *Vibrionales*. Many clades of *Gammaproteobacteria* did not contain any formally cultivated representatives, and only recent advances in cultivation methods, and single cell technologies have started to make up for this. For example, the ubiquitous SAR86 clade, which was originally described in 1995 (Mullins et al., 1995), is associated with a free-living oligotrophic lifestyle in the epipelagic zone (Giovannoni and Vergin, 2012), with distribution patterns similar to that of the SAR11 clade. It has also undergone K-strategist adaptations such as low GC content, presence of a proteorhodopsin gene (Sabehi et al., 2004), and a streamlined metabolism (Dupont et al., 2012; Swan et al., 2013). Another group that is suited to inhabit oligotrophic waters is the group known as Oligotrophic Marine *Gammaproteobacteria* (OMG), which encompasses SAR92, NOR5/OM60, OM182, BD1-7, and KI89A clades (Cho and Giovannoni, 2004). Not as abundant as SAR86, members of the OMG group typically constitute around 3% of the total bacterioplankton community. Perhaps the most thoroughly studied clade in this group is the NOR5/OM60 clade. Cultivated members of this clade were the first marine bacteria that were found to be capable of anoxygenic photosynthesis (Fuchs et al., 2007). Although they were reported from a wide range of marine habitats (Bowman and McCuaig, 2003; Inagaki et al., 2003; Huber et al., 2006), they are generally more abundant in coastal areas than in open ocean settings (Yan et al., 2009). Finally, the marine *Gammaproteobacteria* also contain sulfur oxidizer clades (GSOs), which include SUP05, ARCTIC96BD-19, and Agg47 (Walsh et al., 2009; Swan et al., 2011; Wright et al., 2012; Anantharaman et al., 2013). These clades are strikingly different to the previous oligotrophic clades, such that they are primarily found in dysoxic to anoxic waters of OMZs, as well as in the lower mesopelagic zone (Glaubitz et al., 2009; Lavik et al., 2009; Brown et al., 2014).

In our analysis of ICoMM samples, we observed that the new gammaproteobacterial clades were equally abundant in photic and aphotic zones. Furthermore, after *Alphaproteobacteria*, these *Gammaproteobacteria* clades were still the most abundant among all other phyla (Figure 2). The significant associations to different water body types revealed that these clades occupy low to mid productivity coastal and ocean waters (Table 1). Specifically, *Gammaproteobacteria*.Order1-4 had highest relative abundance in low productivity ocean waters, while *Gammaproteobacteria*.Order1-1, *Gammaproteobacteria*.Order1-5, and *Gammaproteobacteria*.Order9-2 were relatively more abundant in mid productivity ocean waters. On the other hand, *Gammaproteobacteria*.Order9-1 and *Gammaproteobacteria*.Order11-1 were associated with coastal waters (Figure 3).

#### Metabolism predictions

The PICRUSt predictions for orders *Gammaproteobacteria*.Order1-1 to *Gammaproteo bacteria*.Order2 very much resembled those for methanotrophic *Gammaproteobacteria*, such as *Methylomonas methanica* (Boden et al., 2011). Specifically, a hydrogenase that could potentially be involved in hydrogen oxidation, RuBisCo subunits, and methane monooxygenase were predicted for these orders. Further, a nifH was predicted for *Gammaproteobacteria*.Order2. The groups *Gammaproteobacteria*.Order1-1 to *Gammaproteobacteria*.Order1-5 were not associated to any known *Gammaproteobacteria* orders, therefore it is tempting to conclude that marine methanotrophic *Gammaproteobacteria* may be more widely spread than previously believed. In contrast to these predictions, for *Gammaproteobacteria*.Order6, *Gammaproteobacteria*.Order11 and *Gammaproteobacteria*.Order11-1, the PICRUSt predictions pointed toward a heterotrophic lifestyle. For example, complex sugar utilization genes were only found for *Gammaproteobacteria*.Order6, *Gammaproteobacteria*.Order11, and *Gammaproteobacteria*.Order11-1. *Gammaproteobacteria*.Order11-1 was further characterized by the potential to degrade taurine, chitin, and peptides. Given that *Gammaproteobacteria*.Order11-1 is related to *Alteromonadales*, it is likely that it represents a clade similar to NOR5/OM60, found in higher abundance in coastal waters and capable of feeding on phytoplankton storage molecules. *Gammaproteobacteria*.Order6 and *Gammaproteobacteria*.Order11 are related to *Thiotrichales* and *Xanthomonadales*, which are not recognized as typical marine bacteria, but could represent rare members of the community that bloom under certain conditions.

### Verrucomicrobia

#### Phylogeny and taxonomy

*Verrucomicrobia* is a divergent phylum of Bacteria, which includes members of microbial communities from soils, as well as aquatic habitats. They feature interesting characteristics such as genes homologous to eukaryotic tubulins (Jenkins et al., 2002), or methane oxidation in low pH environments (Dunfield et al., 2007). It consists of two classes (*Verrucomicrobiae* and *Opitutae*) with species that have standing in bacterial nomenclature, and another handful of “Candidatus” and not formally described species that have been assigned to putative classes. Isolates originating from the marine environment exist in both classes. After phylogenetic tree reconstruction, and subsequent CTU method application, the sequences were distributed across five orders and 11 families. In general, these groups were not very diverse, with an average of three genera per family (Figure 10; Table S2).

#### Habitat

In the marine environments, *Verrucomicrobia* regularly appear in surveys of polar and temperate zones. It is thought that although only comprising a small fraction of the communities in the water column and sediment, they are widespread (Cottrell and Kirchman, 2000; Schäfer et al., 2000; Madrid et al., 2001; Bowman and McCuaig, 2003; Teske et al., 2011). Furthermore, they have also been identified in marine animals and plants (Weidner et al., 2000; Alain et al., 2002). Finally, the verrucomicrobial sequences recovered from the respective habitats are associated with the so-called subphyla 1 and 4, which correspond to the *Verrucomicrobiae* and *Opitutate* classes, consistent with our full-length 16S rRNA sequence survey.

In our survey of ICoMM samples, we observed that *Verrucomicrobia* were distributed uniformly across different depth zones of neritic and oceanic environments, but with slightly increased relative abundances at or below the mesopelagic zone (Figure 2). Across different marine environments, we observed that they were present across almost all productivity and water column oxygenation types (Figure 3). Low productivity oceanic environment had the highest overall verrucomicrobial relative abundance (Table 1). Other specific patterns that we observed were that *Verrucomicrobia*.Order36 and *Verrucomicrobia*.Order37 were relatively more abundant in coastal waters, and that *Verrucomicrobia*.Order38 and *Verrucomicrobia*.Order39 appeared to be primarily an oceanic clade (Table 1). The relationships of the verrucomicrobial orders with physicochemical parameters varied. For instance, while *Verrucomicrobia*.Order38, 39, and 40 were positively correlated with depth, no correlation could be observed for *Verrucomicrobia*.Order36 and *Verrucomicrobia*.Order37 (Figure 4). Overall, our results are in good agreement with a recent study from Freitas et al. (2012), which was also based on the ICoMM dataset (including sediment) samples and had analyzed the distribution and diversity of the *Verrucomicrobia* phylum. They found that the occurrence of subdivision 4 sequences was highest in surface waters, and that they negatively correlated with depth. In our results, *Verrucomicrobia*.Order36 and *Verrucomicrobia*.Order37 corresponded to the subdivision 4, and were found primarily in coastal waters, but showed no correlation with depth. Additionally, *Verrucomicrobia*.Order38 and *Verrucomicrobia*.Order39, which we believe correspond to “unclassified” *Verrucomicrobia* in the other study, are correlated with nitrate concentration. Freitas et al. reported that this unclassified portion is more frequently found with higher nitrate concentration. Our study additionally reports increasing abundance of certain orders of *Verrucomicrobia* with depth, as supported by another study that has reported that the number of *Verrucomicrobia* increases between 440 and 800 meters water depth (Brown et al., 2009).

#### Metabolism predictions

The PICRUSt predictions (Table S5) suggested a nitrite reductase (nrfA) that could take part in DNRA for all orders except *Verrucomicrobia*.Order40, nifH enzymes for *Verrucomicrobia*.Order36 and *Verrucomicrobia*.Order37, and a number of sulfatases, glycosidases, and proteases that could be involved in degradation of complex organic matter, in numbers comparable to *Bacteroidetes* or *Planctomycetes*. Nitrite reduction would fit well to the observed distribution patters, since, except for *Verrucomicrobia*.Order40, all orders were recovered from anoxic water bodies, thereby providing them with an additional mean of respiration. The nitrogen fixation capability is suspicious, only a recent study reports nitrogen fixation in a *Verrucomicrobia*, which inhabits termite guts (Wertz et al., 2012). On the other hand, nitrogen fixation is predicted for *Puniceicoccaceae* genomic sequences (Markowitz et al., 2012), which *Verrucomicrobia*.Order36 and *Verrucomicrobia*.Order37 are related to. If this is the case, they might utilize nitrogen fixation to supplement organic nitrogen in low nutrient oceanic environments. Finally, the number of complex organic matter degradation enzymes, as well as sulfatases (similar to *Planctomycetes*) matches previous findings, including particle attachment (Eloe et al., 2011) and their positive growth response to the addition of diatom-derived dissolved organic matter in cultures (Landa et al., 2014). Interestingly, compared to other phyla, a large number of genes associated with cellulose and hemicellulose degradation (endoglucanase, alpha-N-arabinofuranosidase, beta-mannosidase, xylose isomerase, xylan 1,4-beta-xylosidase) were predicted for all *Verrucomicrobia* orders. In fact, several studies have also placed marine *Verrucomicrobia* in the role of active polysaccharide degraders in freshwater and marine habitats (Martinez-Garcia et al., 2012; Cardman et al., 2014).

## Conclusions

The current study pursues the culture-independent taxonomic analysis of 92 marine clades that have been so far unrecognized. These clades carry now a nomenclature, a rank and a classification that is compatible with the hierarchical structure proposed by the Bacteriological Code. In addition, the inference of ecological and physiological properties reinforces their taxonomic coherence. We have further demonstrated the usefulness of the CTU approach to give meticulous taxonomic standing to uncultured diversity and to evaluate ranks and classifications of existing taxonomies. In this regard, the remarkable abundance of high taxa (i.e., genus and above) detected, just within the marine microbes, suggests a general lack of criteria for their delineation in phyla like *Actinobacteria, Planctomycetes, Lentisphaerae, Deferribacteres*, and *Proteobacteria*. Therefore, the implementation of this technique into microbiologist's routine foresees an important boost in taxonomy, making it more pragmatic. The inclusion of new CTUs into SILVA taxonomy will guarantee that this alternative taxonomy can be maintained and supervised, as we uncover the tree of life's topology and identify the taxonomically meaningful nodes, with a similar stringency to the canonical taxonomy of taxa with validly published names.

## Author contributions

PYI and FG designed and conducted the research, and analyzed the data. PYA implemented the CTU methodology. JR performed the phylogenetic tree reconstruction and annotations. All authors wrote and reviewed the manuscript.

## Funding

PY and FG were funded by the Max Planck Society. JR was funded by European Research Council Advanced Grant ABYSS (no. 294757).

### Conflict of interest statement

The authors declare that the research was conducted in the absence of any commercial or financial relationships that could be construed as a potential conflict of interest.

## Supplementary material

Figure S1 **World map showing the locations of ICoMM and MIRADA samples used in this study**. The bounding boxes represent Longhurst regions.

Figure S2 **Relative contribution of different phyla to SILVA SSU Ref 111 (all and only newly recognized clades) and ICoMM tag sequences**.

Table S1 **Keywords used for identifying marine water column sequences in SILVA SSU Ref 111 dataset**.

Table S2 **Number of sequences in known and newly recognized marine clades (Sheet 1), references and accession numbers for *known* clades (Sheet 2), taxonomic hierarchy of newly recognized clades (Sheet 3)**.

Table S3 **Metadata and SILVAngs statistics for ICoMM and MIRADA samples (Sheet 1), and definitions of biomes and environments (Sheet 2)**.

Table S4 **Indicator species analysis results for the new clades**. Results are shown for those with a *p*-value of less than 0.05. The light green colored cells indicate that an order can be considered indicator of a given marine environment. LP, low productivity; MP, mid productivity; HP, high productivity.

Table S5 **Average relative abundances of newly recognized clades in different marine environments**.

Table S6 **PICRUSt prediction results for newly recognized clades**. The sheets are organized according to energy (Sheet 1), carbon (Sheet 2), nitrogen (Sheet 3), sulfur (Sheet 4), phosphate (Sheet 5), and other (Sheet 6) metabolism.
