# Supplementary figures and images for: Expanding the World of Marine Bacterial and Archaeal Clades
Source: Front Microbiol. 2016 Jan 8;6:1524. doi: 10.3389/fmicb.2015.01524 (PMC4705458; doi:10.3389/fmicb.2015.01524)

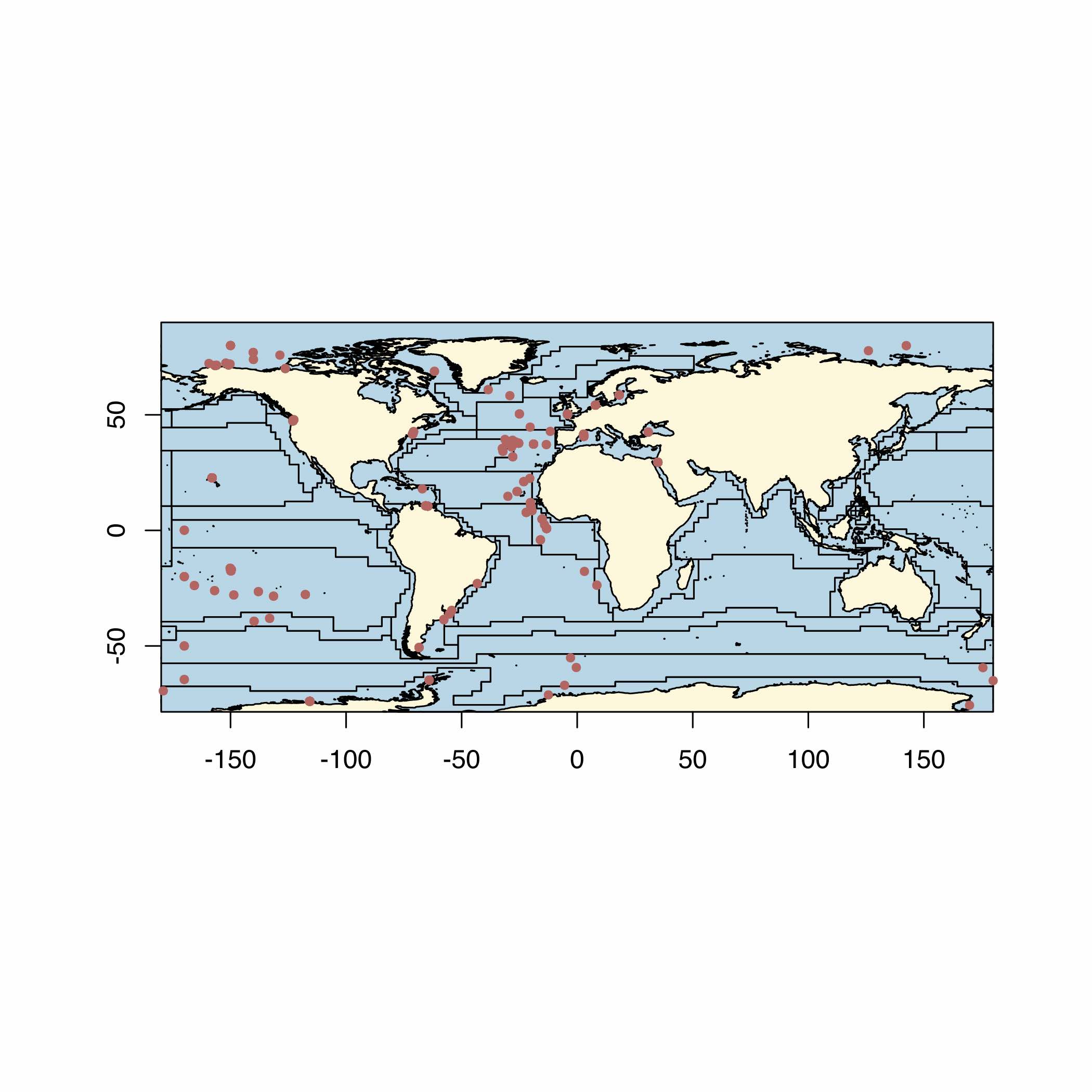

Supplement: Figure S1 — World map showing the locations of ICoMM and MIRADA samples used in this study. The bounding boxes represent Longhurst regions. [file Image1.JPEG]

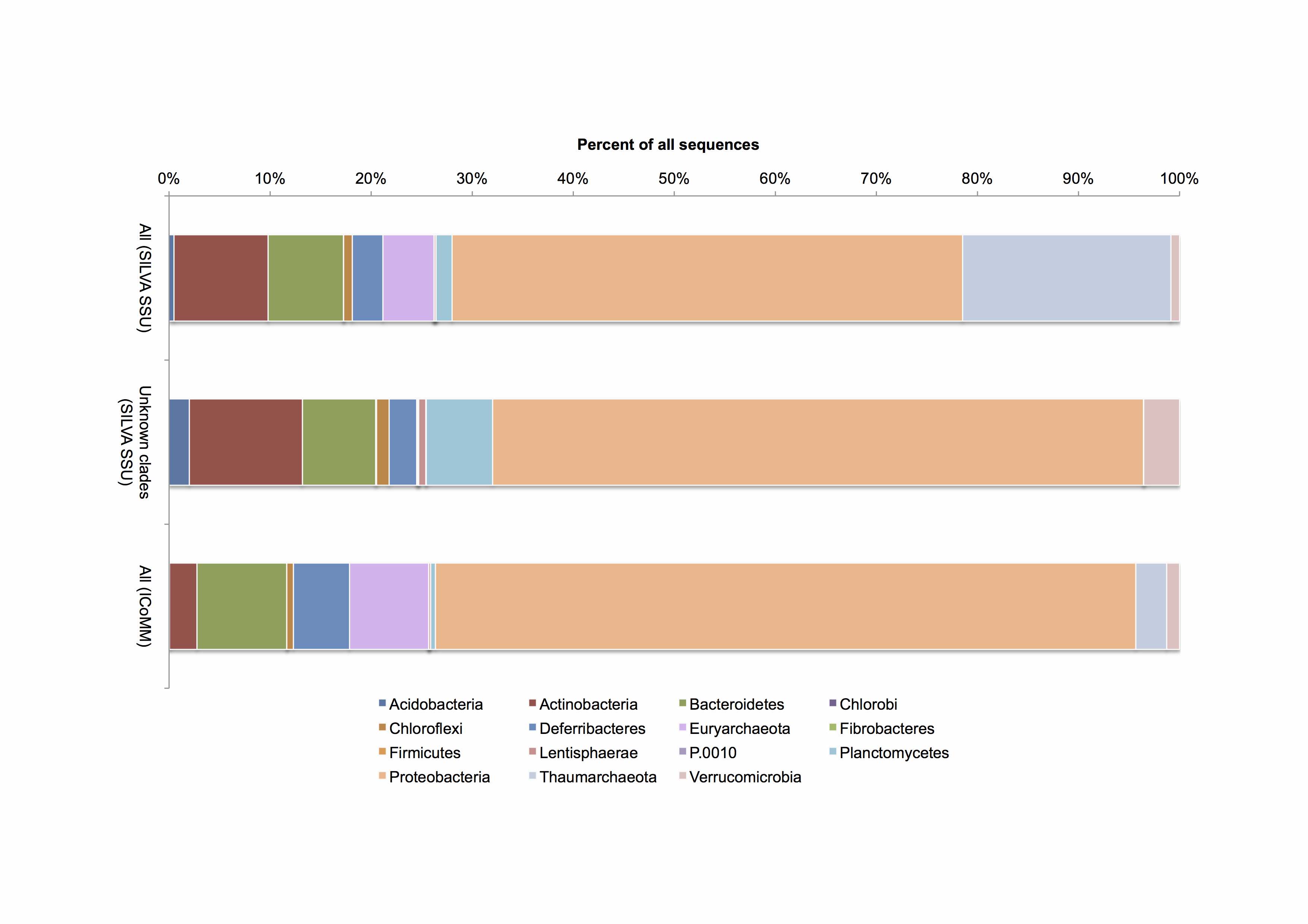

Supplement: Figure S2 — Relative contribution of different phyla to SILVA SSU Ref 111 (all and only newly recognized clades) and ICoMM tag sequences. [file Image2.JPEG]
